# Untargeted Proteomics Identifies Plant Substrates of the Bacterial‐Derived ADP‐Ribosyltransferase AvrRpm1

**DOI:** 10.1002/pld3.70115

**Published:** 2025-11-16

**Authors:** Simranjit Kaur, Thomas Colby, Domenika Thieme, Carsten Proksch, Susanne Matschi, Ivan Matić, Lennart Wirthmueller

**Affiliations:** ^1^ Department Biochemistry of Plant Interactions Leibniz Institute of Plant Biochemistry Halle (Saale) Germany; ^2^ Research Group of Proteomics and ADP‐Ribosylation Signaling Max Planck Institute for Biology of Ageing Cologne Germany

**Keywords:** ADP‐ribosylation, ADP‐ribosyltransferase, bacterial pathogen, plant innate immunity, posttranslational protein modification, proteomics, Type III effector

## Abstract

One class of enzymes that plant pathogens employ to manipulate innate immunity and physiology of the infected cells is host‐targeted ADP‐ribosyltransferases. The bacterial pathogen *Pseudomonas syringae* uses its Type III secretion system to inject several effector proteins with ADP‐ribosyltransferase activity into plant cells. One of them, AvrRpm1, ADP‐ribosylates the plasma membrane‐associated RPM1‐INTERACTING PROTEIN 4 (RIN4) in *Glycine max* and *Arabidopsis thaliana* to attenuate targeted secretion of defense‐promoting compounds. Substrate identification of host‐targeted ADP‐ribosyltransferases is complicated by the biochemical lability of the protein modification during plant protein extraction and in several cases requires prior knowledge of plant immune signaling pathways that are impaired by the ADP‐ribosylating Type III effector. Using the AvrRpm1‐RIN4 pair as a proof of concept, we present an untargeted proteomics workflow for enrichment and detection of ADP‐ribosylated proteins and peptides from plant cell extracts that in several cases provides site resolution for the modification.

## 1 Introduction

The ability to limit activation of plant innate immunity is a prerequisite for successful infection by microbial pathogens (Y. Wang et al. 2022). The Gram‐negative bacterial pathogen *Pseudomonas syringae* pv. *tomato* (*Pst*) injects 29 Type III effectors (T3Es) into the host cell cytoplasm. Collectively, these effectors manipulate host physiology and suppress plant immune responses triggered by recognition of microbe‐associated molecular patterns (MAMPs) (Xin et al. 2016; Cunnac et al. 2011). MAMP recognition by cell surface localized pattern recognition receptors activates pattern‐triggered immunity (PTI) that is particularly effective against attempted invasion by nonadapted pathogens. In contrast, well‐adapted pathogens effectively subvert PTI by translocating effectors into plant cells (Y. Wang et al. 2022). Immunity against such adapted pathogens in plants is conferred by a second class of intracellular immune receptors, nucleotide‐binding domain leucine‐rich repeat receptors (NLRs), in a cultivar‐specific manner (Saur et al. 2021). This form of NLR‐mediated resistance to specific pathogen isolates is known as effector‐triggered immunity (ETI) (Saur et al. 2021). NLRs detect pathogen effectors either directly or they form protein complexes with virulence targets of effectors and sense effector‐mediated manipulation of the “guarded” plant proteins (J. Wang et al. 2019; Martin et al. 2020). Therefore, the outcome of an infection attempt by an adapted pathogen depends on both the set of effectors delivered by the pathogen and the NLR repertoire of the plant. In the absence of NLR‐mediated recognition, effectors promote pathogen virulence (Y. Wang et al. 2022). In contrast, in interactions where at least one effector is recognized by an NLR, the plant rapidly activates ETI that limits further pathogen spread and often involves localized programmed cell death at the site of infection (Saur et al. 2021).

Many bacterial T3Es are host‐targeted enzymes that manipulate plant proteins by posttranslational modifications to perturb activation of plant immunity (Y. Wang et al. 2022). Among the T3Es that *Pst* strain DC3000 injects into plant cells are several ADP‐ribosyltransferases that modify host proteins by transfer of the ADP‐ribose (ADPr) moiety from nicotinamide adenine dinucleotide (NAD^+^) onto amino acid side chains (Fu et al. 2007; Y. Wang et al. 2010; Aung et al. 2020; Suskiewicz et al. 2023). The T3E HopF2 ADP‐ribosylates Arabidopsis MITOGEN‐ACTIVATED PROTEIN KINASE KINASE 5 (MKK5), thereby interfering with signal transduction from activated pattern recognition receptors to the nucleus (Y. Wang et al. 2010). HopF2 can also ADP‐ribosylate Arabidopsis RIN4 in vitro (Y. Wang et al. 2010). Overexpression of a HopF2 transgene in Arabidopsis prevents cleavage of RIN4 by another T3E, the cysteine protease AvrRpt2 (Wilton et al. 2010). This indicates that HopF2 ADP‐ribosylates residues in the nitrate‐induced (NOI) domains of RIN4 that are essential for cleavage by AvrRpt2. RIN4 is targeted by additional T3Es from different *P. syringae* pathovars, and it is guarded against effector‐induced manipulation by the NLRs RESISTANCE TO *P. SYRINGAE* PV MACULICOLA 1 (RPM1) and RESISTANT TO *P. SYRINGAE* 2 (RPS2) in the Col‐0 accession (Belkhadir et al. 2004). The T3E AvrRpm1 from *P. syringae* pv. *maculicola* ADP‐ribosylates RIN4 proteins from Arabidopsis and soybean at two positions in their N‐ and C‐terminal NOI domains (Redditt et al. 2019). In addition to RIN4, AvrRpm1 also modifies several sequence‐related Arabidopsis NOI proteins by ADP‐ribosylation (Redditt et al. 2019). Following identification of the ADP‐ribosylation sites on soybean RIN4b by proteomics, Redditt et al. (2019) predicted, based on sequence homology, that AvrRpm1 modifies Arabidopsis RIN4 on N11 and D153. RPM1 is activated by modifications in the C‐terminal NOI (C‐NOI) of RIN4 (Chung et al. 2011). A RIN4 D153A mutant variant that can no longer be ADP‐ribosylated in the C‐NOI attenuates AvrRpm1‐induced activation of RPM1 (Redditt et al. 2019).

Previously, different approaches have been employed to identify substrates of plant‐targeted bacterial ADP‐ribosyltransferases. Fu et al. (2007) used ADP‐ribosylation assays with ^32^P‐NAD^+^ in plant cell extracts in combination with anion exchange chromatography and two‐dimensional gel electrophoresis to identify the RNA‐binding protein GRP7 as a substrate of the T3E HopU1 from *Pst* DC3000. In other cases, prior knowledge about T3E interference with distinct immune signaling pathways, protein interactors, and/or subcellular localization of bacterial ADP‐ribosyltransferases in plant cells has aided in the identification of potential host targets (Y. Wang et al. 2010; Wilton et al. 2010; Zhou et al. 2014; Aung et al. 2020; Wu et al. 2024). For three plant substrates, ADPr‐modification sites have been determined by targeted in vitro ADP‐ribosylation assays in combination with site‐directed mutagenesis (Fu et al. 2007; Y. Wang et al. 2010; Wu et al. 2024). Detection of ADP‐ribosylation sites by proteomics following immunoprecipitation of presumed host target proteins from plant cell extracts has only recently been established, and this approach so far relies on prior identification of effector targets and overexpression of epitope‐tagged proteins (Redditt et al. 2019; Yoon et al. 2022). Given that putative host‐targeted ADP‐ribosyltransferases are not limited to *P. syringae* but are also present in other bacterial and fungal plant pathogens (Seong and Krasileva 2021), we sought to establish proteomics methods that can aid in the direct detection of ADP‐ribosylated proteins from plant protein extracts. Here, we present a proteomics workflow that can identify ADP‐ribosylated proteins from transgenic lines expressing bacterial ADP‐ribosyltransferases by comparative proteomics and provide site resolution for the ADP‐ribosylated peptides.

## 2 Materials and Methods

### 2.1 Experimental Design and Statistical Rationale

**FIGURE 1:**
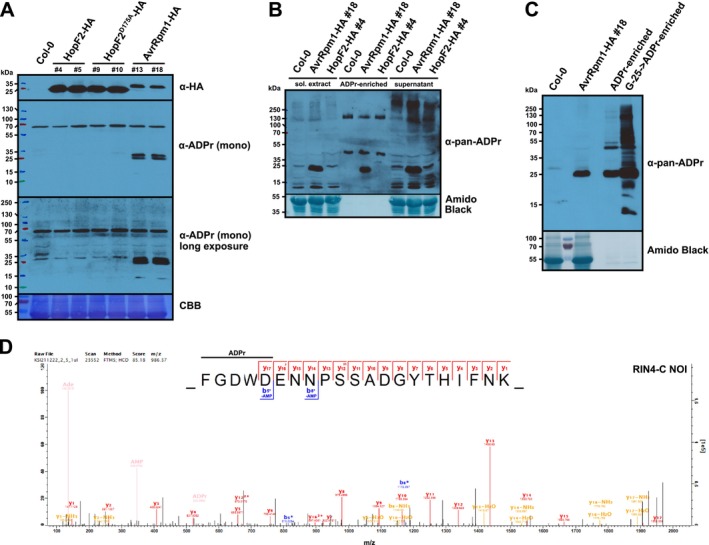
ADP‐ribosylated substrates of AvrRpm1 but not HopF2 can be detected by immunoblots, enriched by the engineered Af1521 Macro domain, and identified by untargeted proteomics. (A) Detection of AvrRpm1 substrates by an α‐ADPr immunoblot. Protein extracts from Dex‐treated Col‐0 wild type and the indicated transgenic lines were separated by SDS‐PAGE, electroblotted onto PVDF membrane and probed with α‐HA and α‐mono‐ADPr antibodies. CBB = Coomassie brilliant blue–stained membrane as loading control. (B) Enrichment of ADP‐ribosylated proteins from the indicated Arabidopsis lines by the engineered Af1521 Macro domain and detection in an α‐pan‐ADPr immunoblot. Approximately 15% of the ADPr‐enriched fraction was loaded onto the gel. “sol. extract”= soluble protein extract. The membrane was stained with Amido black as loading control. This experiment was repeated twice with similar results. (C) Depletion of potentially competing nucleotide analogs from plant extracts prior to the Af1521 affinity purification facilitates enrichment of ADP‐ribosylated proteins. “Col‐0” and “AvrRpm1‐HA #18” indicate the respective protein extracts before ADPr‐enrichment. “ADPr‐enriched” = AvrRpm1 sample, direct pulldown with the Af1521 Macro domain. “G‐25‐>ADPr‐enriched” = AvrRpm1 sample, preclearing by G‐25 Sepharose followed by pulldown with the Af1521 Macro domain. ADP‐ribosylated proteins were detected as in (B). (D) HCD MS^2^ spectrum of the ADP‐ribosylated peptide FGDWDENNPSSADGYTHIFNK from the RIN4 C‐NOI identified by untargeted proteomics (*z* = 3, mass = 2954.09 Da). Unmodified fragment ions and fragment ions with a neutral loss of AMP are indicated in the sequence logos. The horizontal line designates the possible sequence window for ADP‐ribosylation. The ADPr marker ions are shown in beige. Asterisks indicate b‐ions with a neutral loss of AMP (Δ347.06 Da).

The immunoblots presented in Figure 1A–C are representative of three sets of samples derived from independent experiments. For Dataset S1, samples from four independent experiments were analyzed by LC–MS/MS. Wild‐type Col‐0 plants served as a control for T3E‐dependent ADP‐ribosylation. To minimize biological and sample variation during method optimization, Datasets S2 and S3 were derived from one biological dexamethasone (Dex)‐treated AvrRpm1 sample that was subjected to ADPr‐enrichment using 1, 2.5, or 5 μg of the Af1521 Macro domain. The derived peptide samples were first analyzed by the standard LC–MS/MS method (Dataset S2) and immediately afterwards profiled again using the “triggering” method (Dataset S3) to allow for a direct comparison of the two methods. The comparison of proteins enriched by the functional Af1521 Macro domain but not by the G42E variant (Dataset S4) was derived from three independent Dex‐AvrRpm1 samples. Data from Datasets S1 and S4 were combined in Table 1 to identify potential AvrRpm1 substrates supported by evidence from both approaches. AvrRpm1‐dependent ADP‐ribosylation of MAMI (Figure 3B) was tested in three independent experiments, one of which was profiled by LC–MS/MS (Dataset S5) to confirm that the α‐pan‐ADPr immunoblot signal is indeed due to ADP‐ribosylation. The ADP‐ribosylated PLDGAMMA3 peptide (Dataset S6) was identified in two out of three experiments. For Dataset S7, samples from two independent experiments were analyzed by LC–MS/MS. For proteomics analyses, a decoy database search was performed to determine the peptide identification false discovery rate (FDR). Peptides with a score surpassing the FDR threshold of 0.01 (*q* value < 0.01) were considered positive identifications. A detailed description of the proteomics methods is provided below and in Dataset S8.

**TABLE 1:** Proteins specifically identified in Af1521 Macro‐enriched AvrRpm1‐HA samples (≥ 10 collective MS^2^ scans from four experiments) are not or not strongly enriched by the Af1521 G42E mutant variant (based on three experiments). The last column reports significant quantitative differences between the AvrRpm1 and Col‐0 samples based on LFQ and a FDR of 0.05. Proteins that were excluded from LFQ‐based quantification in MaxQuant are indicated by “not quantified” (n. q.).

AGI identifier	Description	MS^2^ Col‐0	MS^2^ AvrRpm1	MS^2^ HopF2	MS^2^ counts Af1521/G42E	Sign. by LFQ
AT3G25070.1	RIN4	0	40	0	42/8	Yes
AT3G45780.2	PHOT1	0	31	0	29/1	n. q.
AT4G11840.2	PLDGAMMA3	0	22	0	17/0	Yes
AT5G54110.1	MAMI	0	20	0	15/0	n. q.
AT3G07195.1	NOI11	0	17	0	12/0	n. q.
AT3G24550.1	PERK1	0	16	0	12/0	Yes
AT1G70940.1	PIN3	0	13	0	5/0	n. q.
AT2G02170.5	Remorin family protein	0	12	0	13/0	n. q.
AT3G63260.1	MRK1	0	12	0	10/0	n. q.
AT4G21450.1	PapD‐like superfamily protein	0	12	0	11/0	n. q.
AT2G04410.1	NOI12	0	10	0	8/1	n. q.
AT4G13350.2	NSP INTERACTING GTPASE	0	10	0	12/0	n. q.

### 2.2 Plant Material and Growth Conditions

The transgenic lines expressing Dex‐inducible AvrRpm1‐HA and HopF2‐HA have been described (Mackey et al. 2002; Wilton et al. 2010). Transgenic Arabidopsis lines expressing a Dex‐inducible HopU1‐3xFlag construct were generated by transforming Col‐0 plants with pTA7001/des/HopU1‐3xFlag. Arabidopsis plants were grown in controlled environment chambers with a 10‐h light period and 22°C/18°C day/night temperatures. To induce expression of T3Es, 4‐week‐old plants were sprayed with a solution containing 20‐μM Dex and 0.01% Silwet‐77, and leaves were harvested 16–18 h later. *Nicotiana benthamiana* plants were cultivated under long day (16‐h light, 8‐h dark) conditions at 22°C and approximately 30% relative humidity in a greenhouse under supplementary light from tungsten lamps at approximately 200 μmol/s/m^2^.

### 2.3 DNA Constructs

The G42E mutation in the engineered Af1521 Macro domain was introduced by site‐directed mutagenesis in pGEX6P‐1 (Nowak et al. 2020). The functional (K35E and Y145R) and nonfunctional (K35E, G42E, and Y145R) variants were cloned into KpnI/HindIII‐linearized pOPINF (Berrow et al. 2007) by Gibson cloning to create His6‐GST‐Macro constructs. *Escherichia coli* expression constructs for HopF2 and MKK5 were created by cloning the coding sequences into KpnI/HindIII‐linearized pOPINF (Berrow et al. 2007). The MAMI, PHOT1, and AvrRpm1 coding sequences as well as the genomic sequence of PLDGAMMA3 were cloned into pENTR4 by Gibson cloning. The N‐terminally tagged StrepII‐3xHA‐MAMI construct was created by a Gateway LR reaction with pENS‐HS‐GW. The C‐terminally tagged PHOT1‐3xHA‐StrepII and PLDGAMMA3‐3xHA‐StrepII constructs were created by Gateway LR reactions with pXCSG‐GW‐HS. AvrRpm1 and HopU1 were fused to a 3xFlag tag by a Gateway LR reaction with pTA7001/des/3xFlag (Li et al. 2013). Binary vectors were transformed into *Agrobacterium tumefaciens* strain GV3101 pMP90^RK^ (pENS and pXCSG) or GV3101 pMP90 (pTA7001).

### 2.4 Agrobacterium‐Mediated Transient Expression

*A. tumefaciens* strains were grown on selective YEB plates and resuspended in infiltration buffer (10‐mM MgCl_2_, 10‐mM MES‐KOH pH 5.6, and 150‐μM acetosyringone). Each strain was mixed with *A. tumefaciens* strain GV3101 pMP90 expressing the silencing suppressor 19K at a ratio of 1:1. The cultures were infiltrated into leaves of 4‐ to 5‐week‐old *N. benthamiana* plants using a needleless syringe. After 2 days, AvrRpm1 expression was induced by spraying infiltrated leaves with Dex as above, and leaf material for protein extraction was harvested 8 h later.

### 2.5 Plant Protein Extraction and Detection by Immunoblots

Protein extracts were prepared by grinding plant leaf material in liquid N_2_ to a fine powder followed by resuspension in extraction buffer (50‐mM Tris–HCl, 150‐mM NaCl, 10% (v/v) glycerol, 1‐mM EDTA, 5‐mM DTT, 1 × protease inhibitor cocktail [Sigma‐Aldrich #P9599], 10‐μM MG132 [Merck], and 0.2% NP‐40, pH 7.4) at a ratio of 2‐mL buffer per 1‐g leaf material. The amount of leaf material was 1.5–2 g for *N. benthamiana* and 2–4 g for Arabidopsis. Samples for direct comparisons had the same amount of starting material. Crude protein extracts were centrifuged at 20,000 × *g*/4°C/20 min, and the supernatant was either used for SDS‐PAGE or subjected to ADPr‐enrichment with the Af1521 Macro domain. For immunoblots, proteins were separated by SDS‐PAGE and electroblotted onto PVDF membrane. The membrane was blocked with 5% nonfat dry milk in TBST for 1 h at RT. Proteins were detected using α‐HA 3F10 antibody (Merck, 11867423001, 1:4000), α‐Flag M2 antibody (Merck, F3165, 1:1000), α‐pan‐ADPr‐binding reagent (Merck, MABE1016, 1:4000), α‐mono‐ADPr AbD33204 (Bio‐Rad, 1:2000), or α‐ADPr E6F6A (Cell Signaling, #83732, 1:5000) in 2.5% nonfat dry milk in TBST overnight at 4°C. After three wash steps in TBST, membranes were incubated with HRP‐coupled secondary antibodies (Merck, 1:20,000). The membranes were washed four times in TBST, and proteins were detected using chemiluminescence substrates ECL Prime Western Blotting Detection Reagent (Cytiva) or SuperSignal West Femto Maximum Sensitivity Substrate (Thermo Fisher) and X‐ray films (Figures 1 and S6) or a Fusion Fx imager (Vilber) (Figures 3 and S5).

### 2.6 Enrichment of ADP‐Ribosylated Proteins

Per affinity purification, 15‐μL Glutathione‐Sepharose 4B resin (Merck) was washed in 50‐mM Tris–HCl pH 7.5, 50‐mM NaCl, and 1‐mM DTT and pelleted by centrifugation (500 × *g*, RT, 1 min); 1, 2.5, or 5 μg of GST‐Macro (or G42E) were added in a total volume of 1‐mL wash buffer, and the tubes were rotated for 1 h at 4°C. The resin was pelleted as above and washed twice with 0.8‐mL plant extraction buffer (see Section 2.5). Cold plant extract was either added directly to the resin (Dataset S1) or precleared by filtration over HiTrap desalting columns (see below, Datasets S2–S7). The samples were rotated for 2 h at 4°C and washed three times with 1‐mL cold plant extraction buffer. The proteins were eluted from the resin by adding SDS sample buffer and incubation at 75°C for 5 min.

### 2.7 Preclearing of Samples Prior to Enrichment of ADP‐Ribosylated Proteins

To deplete nucleotides that also can bind to the Af1521 Macro domain (Karras et al. 2005), 2–3.5 mL of plant protein extract was applied to three stacked 5‐mL HiTrap desalting columns (Cytiva) using a manual syringe. The columns were connected to an FPLC system and eluted with plant protein extraction buffer at a flow rate of 1.2 mL/min. Fifteen fractions of 0.5 mL were collected while monitoring absorbance at 280 nm, and the protein fraction was determined as the first peak eluting from the column.

### 2.8 Sample Preparation for LC–MS/MS

Protein samples were separated by 12% SDS‐PAGE until the running front had migrated 1 cm into the separating gel. The gels were stained with Coomassie, and the protein area was cut from the gel. Proteins were in‐gel digested with trypsin and desalted as described by Majovsky et al. (2014).

### 2.9 Acquisition of Raw Datasets by Higher Energy Collisional Dissociation (HCD) Fragmentation

Peptides were analyzed with five different MS methods on a Fusion Lumos Tribrid mass spectrometer (Thermo Fisher Scientific), summarized in Dataset S8. Dried peptides were dissolved in 5% acetonitrile and 0.1% trifluoric acid and injected into an EASY‐nLC 1200 liquid chromatography system (Thermo Fisher Scientific). Peptides were separated using liquid chromatography C_18_ reverse phase chemistry employing a 120‐min gradient increasing from 1% to 24% acetonitrile in 0.1% FA (MS Method 1) or from 1% to 36% acetonitrile in 0.1% FA (MS Methods 2–5) and a flow rate of 250 nL/min. Eluted peptides were electrosprayed on‐line into the Fusion Lumos Tribrid with a spray voltage of 2.0 kV and a capillary temperature of 305°C. Peptides (MS^1^) were detected in the Orbitrap with the following settings: resolving power: 120,000; scan range *m/z* 300–1500; S‐Lens RF 30%; AGC target standard, max injection time (IT) on auto mode, microscans: 1. Dynamic exclusion duration was set for 60 (MS Method 1) or 30 s (MS Methods 2–5). MS/MS peptide sequencing was performed using a Top15 DDA scan strategy (MS Method 1), or peptides were selected for a 1‐s time window in between master scans in a data‐dependent mode (MS Methods 2–5) with HCD at 30% NCE, to be detected in the Orbitrap with a resolution of 30,000. For differences in AGC target and maximum IT mode between different methods, see Dataset S8. Based on total ion chromatogram intensities from premeasurements, the volume of sample loaded onto the LC was adjusted to reach the maximal capacity of the column. Different targeted mass exclusion lists containing the most prominent peptide *m/z* ratios for the GST‐Af1521 Macro domain and, where appropriate, GSTs from *Arabidopsis thaliana* were used for MS Methods 2, 3, and 5 (see Dataset S8). For Methods 3–5, an additional MS^2^ scan with an isolation window of 1.3 was triggered by the presence of the diagnostic peak of adenine at 136.062 Da in a precursor's fragmentation spectrum. Orbitrap detection occurred at a resolution of 60,000 with a defined first mass of 120 *m/z*. The normalized AGC target of the triggered scan was set to 1000%, and the maximum IT was 1800 ms.

### 2.10 Identification of Proteins and Peptides

Peptides and proteins were identified using MaxQuant software v2.1.3.0 (Tyanova, Temu, Sinitcyn, et al. 2016). For Datasets S1–S4 and S7, the data were searched against the *A. thaliana* Araport11 proteome database downloaded from https://www.arabidopsis.org/download/ on 03.01.2022 and the sequences of the GST‐Af1521 Macro domains. For Datasets S5 and S6, peptides and proteins were identified by searches against the *N. benthamiana* NbDE proteome database (Kourelis et al. 2019) downloaded on 22.12.2022. In all searches, a FDR of 0.01 was applied for PSM, protein, and site decoy fraction. The minimal peptide length was seven amino acids, the enzyme specificity was set to Trypsin/P, and two missed cleavages were tolerated. Carbamidomethylation of cysteine (C) was set as a fixed modification. Variable modifications were oxidation of methionine (M), acetylated protein N‐termini, and ADP‐ribosylation of aspartic acid (D), glutamic acid (E), asparagine (N), lysine (K), arginine (R), serine (S), cysteine (C), and histidine (H). For PLDGAMMA3 expressed in *N. benthamiana* (Dataset S6), ADP‐ribosylation of glutamine (Q) was added. Neutral losses of adenine (135.0545 Da), AMP (347.0631 Da), adenosine H_2_O (249.0862 Da), ADP (427.0294 Da), and ADPr (541.0611 Da) were included for data analysis. Adenine^+^ (136.0618 Da), AMP^+^ (348.0704 Da), and ADPr^+^ (542.0684 Da) were used as diagnostic peaks for ADP‐ribosylation. For the analysis of ADP‐ribosylated peptides in MaxQuant, we (i) applied an Andromeda score of > 80 and (ii) manually inspected MS^2^ spectra for ADPr marker ions and neutral losses characteristic of ADP‐ribosylation.

### 2.11 Label‐Free Quantification (LFQ) of Proteins

Proteins were quantified by LFQ as implemented in MaxQuant with default settings. The “Match between runs” option was enabled if the samples were run on the same column (Datasets S2 and S3) but was disabled for Datasets S1 and S4 as not all replicates were analyzed on the same LC column. For quantification in Perseus v2.0.7.0 (Tyanova, Temu, and Cox 2016), the ProteinGroups.txt file from MaxQuant was imported and processed as follows. Potential contaminants, reverse hits, and proteins only identified by site were removed. LFQ intensities were log_2_‐transformed, and only those proteins that had assigned LFQ values in all samples of at least one group were kept. Missing values were replaced by zero, and statistical analysis and visualization were based on the volcano plot function in Perseus. Pearson correlations were calculated with the multiscatter plot function of Perseus.

## 3 Results

We used transgenic lines that express AvrRpm1‐HA or HopF2‐HA under control of a Dex‐inducible promoter to assess if ADP‐ribosylated endogenous RIN4 protein (24 kDa) can be detected by immunoblot analysis. Samples from two independent AvrRpm1‐expressing lines showed two bands between 25 and 35 kDa when probed with an antibody specific to mono‐ADP‐ribosylation (Bonfiglio et al. 2020). In contrast, the antibody did not detect prominent bands in the two lines expressing HopF2‐HA (Figure 1A). Even upon longer exposure, the band pattern of the HopF2 samples looked similar to those from Col‐0 and two lines carrying a transgene encoding the enzymatically inactive HopF2 D175A variant. As shown by the α‐HA immunoblot in Figure 1A, both HopF2‐HA protein variants were expressed, suggesting that HopF2 substrates might be proteins of low abundance or that they were not efficiently extracted from plant tissue by our method. Next, we used the engineered version of the Af1521 Macro domain (Nowak et al. 2020) to enrich ADP‐ribosylated proteins from plant extracts (Figure 1B). As the Af1521 Macro domain can also bind to the terminal ADPr of poly(ADPr) chains, the signal on the immunoblot probed with α‐pan‐ADPr‐binding reagent might represent a mixture of mono‐ and poly‐ADP‐ribosylated proteins. By LC–MS/MS, we identified 950 protein groups that were supported by at least two MS^2^ scans. The overlap between the three sample groups was 81%. RIN4 was detected with 40 MS^2^ scans in four samples from an AvrRpm1‐expressing line but not in Col‐0 or HopF2 samples (Table 1 and Dataset S1). Table 1 lists an additional 11 proteins, including the RIN4 homologs NOI11 and NOI12, that were identified with ≥ 10 MS^2^ scans in ADPr‐enriched AvrRpm1 samples but not in the Col‐0 or HopF2 pulldowns. Out of the 113 protein groups that were assigned LFQ values in MaxQuant in all four experiments, only RIN4, PHOSPHOLIPASE D GAMMA 3 (PLDGAMMA3), and PROLINE EXTENSIN‐LIKE RECEPTOR KINASE 1 (PERK1) were identified as significantly different from Col‐0 samples with a FDR of 0.05 (Figure S1A). The comparison of ADPr‐enriched HopF2 versus Col‐0 samples by LFQ did not reveal any differentially abundant proteins (Figure S1B). The differences between LFQ and quantification based on MS^2^ scans are explained by the fact that 9 out of the 12 proteins from Table 1 were not quantifiable by LFQ. In summary, this comparison provided a set of candidate proteins that might be ADP‐ribosylated by AvrRpm1, but it did not detect any ADP‐ribosylated peptides of RIN4 or other proteins. We found that a depletion of potentially competing nucleotide analogs (Karras et al. 2005) by a Sephadex G‐25 column resulted in stronger enrichment by the Af1521 Macro domain (Figure 1C) and included this separation step in the protocol for further ADPr‐enrichments.

To optimize detection of ADP‐ribosylated peptides, we focused on substrates of AvrRpm1. Untargeted proteomics of the precleared and ADPr‐enriched AvrRpm1 sample detected ADP‐ribosylated peptides of the NOI sequences from NOI4 (AT5G55850.3), NOI12 (AT2G04410.1), and the N‐NOI and C‐NOI sequences of RIN4 (Dataset S2 and Table S1). Although the marker ions of ADPr fragmentation (Ade^+^ 136.06, AMP^+^ 348.07, and ADPr^+^ 542.07 Da) and MaxQuant (Tyanova, Temu, Sinitcyn, et al. 2016) Andromeda scores between 68 and 101 clearly identified these peptides as ADP‐ribosylated (Figure 1D and Table S1), the localization probability for ADPr‐modification sites was often below 80% and therefore ambiguous. Next, we used a data‐dependent “triggering” method that initiates an MS^2^ scan with an elevated threshold for automatic gain control, higher resolution, and longer IT upon detection of the 136.06‐Da Adenine^+^ fragment of ADPr that is typically an intense fragment ion from ADP‐ribosylation sites (Rosenthal et al. 2015; Geiszler et al. 2023) (Dataset S3). In total, Datasets S2 and S3 identified 399 protein groups that were supported by at least two MS^2^ scans. The overlap between the two datasets was 75%. A subset of 140 protein groups was detected in all three experiments of at least one dataset, showed pairwise Pearson correlations of 0.85–0.98, and could be compared by LFQ in MaxQuant. The quantified protein groups between Datasets S2 and S3 differed in seven protein groups that were only quantified in Dataset S3. Three of those, a RING/U‐box superfamily protein (AT1G47570), RIBOSOMAL LARGE SUBUNIT 4 (AT5G02870), and ATSAP18 (AT2G45640), were only detected by the “triggering” method (Figure S2A,B). However, for these three proteins, no ADP‐ribosylated peptides were detected. Overall, we consider the two datasets comparable.

**FIGURE 2:**
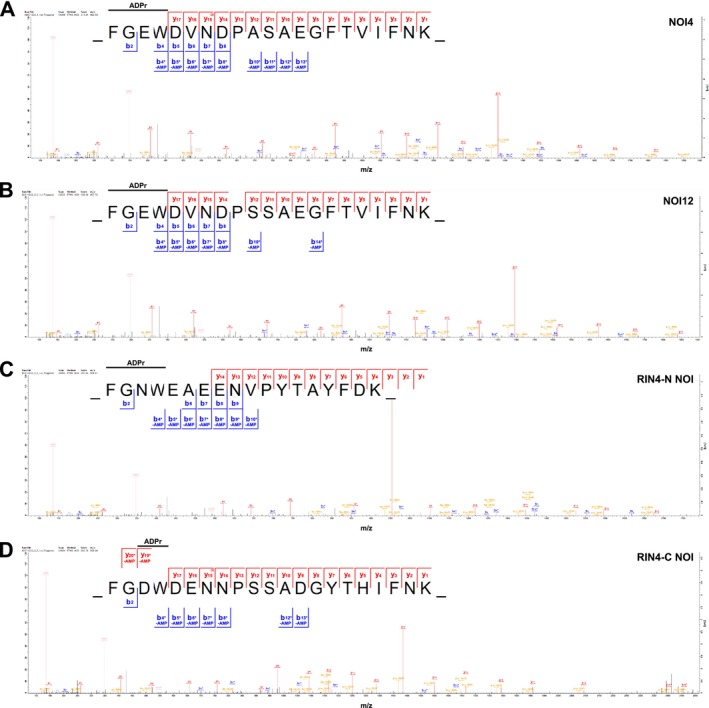
ADPr‐modification sites on AvrRpm1 substrates revealed by the “triggering” HCD fragmentation method. Panels (A)–(D) show MS^2^ spectra of ADP‐ribosylated peptides from NOI4, NOI12, and the RIN4 N‐NOI and C‐NOI sequences. Unmodified fragment ions and fragment ions with a neutral loss of AMP are indicated in the sequence logos. The horizontal line designates the possible sequence window for ADP‐ribosylation. (A) NOI4‐derived peptide FGEWDVNDPASAEGFTVIFNK (*z* = 3, mass = 2883.17 Da). (B) NOI12‐derived peptide FGEWDVNDPSSAEGFTVIFNK (*z* = 3, mass = 2899.14 Da). (C) RIN4 N‐NOI‐derived peptide FGNWEAEENVPYTAYFDK (*z* = 3, mass = 2720.01 Da). (D) RIN4 C‐NOI‐derived peptide FGDWDENNPSSADGYTHIFNK (*z* = 3, mass = 2954.09 Da). The ADPr marker ions are shown in beige. Asterisks indicate ions with a neutral loss of AMP (Δ347.06 Da).

Differences between the two MS^2^ methods became apparent in a qualitative comparison of the detected ADP‐ribosylation sites (Figure S2C and Table S1). The “triggering” method used for Dataset S3 resulted in more informative ion series, including additional neutral losses of AMP (Δ347.06 Da) (Figure 2 and Dataset S3). Consequently, the ADPr localization probabilities improved to > 80%, and from three LC–MS/MS runs, we could identify at least one MS^2^ spectrum for each modified RIN4/NOI peptide with a localization score of > 99.5%. Based on these data and the assumption that ADP‐ribosylation does not occur on phenylalanine (F), glycine (G), or tryptophan (W) (Rack et al. 2020), the NOI4 protein was ADP‐ribosylated on E29 (Figure 2A), and for NOI12, we observed ADP‐ribosylation on the sequence‐equivalent position E13 (Figure 2B). RIN4 was ADP‐ribosylated on N11 (N‐NOI, Figure 2C) and D153 (C‐NOI, Figure 2D). Based on a limited comparison of performing the enrichment with different amounts of the Af1521 Macro domain (1, 2.5, or 5 μg) (Table S2), we used 2.5 μg for subsequent experiments. In conclusion, based on the AvrRpm1‐RIN4 proof‐of‐concept experiments, our untargeted method robustly identified RIN4 and RIN4 homologs as *in planta* substrates of AvrRpm1 and allowed mapping the ADP‐ribosylation sites with high confidence.

**FIGURE 3:**
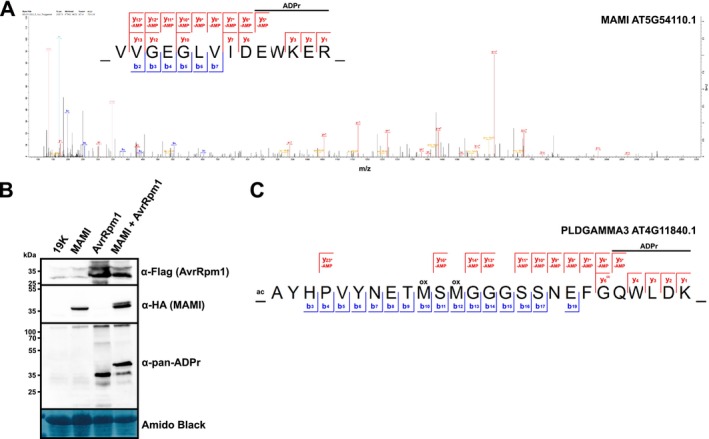
AvrRpm1 ADP‐ribosylates Arabidopsis MAMI and PLDGAMMA3. (A) HCD MS^2^ spectrum of the ADP‐ribosylated MAMI peptide VVGEGLVIDEWKER (*z* = 3, mass = 2168.92 Da). The ADPr marker ions are shown in beige. Unmodified y‐ions and y‐ions with a neutral loss of AMP are indicated above the sequence logo. The horizontal line designates the possible sequence window for ADP‐ribosylation. Asterisks indicate ions with a neutral loss of AMP (Δ347.06 Da). (B) Detection of ADP‐ribosylated StrepII‐HA‐MAMI transiently coexpressed with AvrRpm1‐Flag in *Nicotiana benthamiana*. Protein extracts were separated by SDS‐PAGE, electroblotted onto PVDF membrane and probed with α‐HA, α‐pan‐ADPr, or α‐Flag antibodies. The membrane was stained with Amido black as loading control. “19K” indicates a sample from leaves expressing only the silencing suppressor 19K. The results are representative of three independent experiments. Images of the complete membranes are shown in Figure S3. (C) ADP‐ribosylated peptide AYHPVYNETMSMGGGSSNEFGQWLDK (*z* = 3, mass = 3519.31 Da) from PLDGAMMA3‐HA‐StrepII coexpressed with AvrRpm1‐Flag in *N. benthamiana*. Unmodified y‐ions and y‐ions with a neutral loss of AMP are indicated above the sequence logo. The horizontal line designates the possible sequence window for ADP‐ribosylation. Asterisks indicate ions with a neutral loss of AMP (Δ347.06 Da).

While MaxQuant reports several ADP‐ribosylated peptides in addition to those from NOI4, NOI12, and RIN4, their MS^2^ spectra overall had lower Andromeda scores and lacked the ADP‐ribosylation marker ions (Dataset S3). These spectra were also characterized by a higher signal‐to‐noise ratio, and in many cases, the incomplete b‐ and y‐ion series did not cover the assumed ADP‐ribosylation site. Therefore, the data did not provide sufficient evidence for ADP‐ribosylation. One exception was a peptide from the MEMBRANE‐ASSOCIATED MANNITOL‐INDUCED (MAMI, AT5G54110.1) protein (Figure 3A) that was identified in one of the three LC–MS/MS runs. In this case, an almost complete y‐ion series with neutral losses of AMP together with the Ade^+^ and AMP^+^ marker ions indicated ADP‐ribosylation of peptide VVGEGLVIDEWKER (236–249) with the highest localization probabilities for E245 or E248 (underlined, 33%, respectively). If MAMI is ADP‐ribosylated by AvrRpm1, the protein should be enriched compared to controls in our ADPr affinity purification. Indeed, we identified MAMI with 20 MS^2^ scans in ADPr‐enriched samples of Dex‐treated AvrRpm1 plants but not in Dex‐treated HopF2 or Col‐0 controls (Table 1 and Dataset S1). Overexpression of AvrRpm1 may stabilize or elevate MAMI protein levels, which could result in higher carryover of MAMI‐derived peptides during the affinity enrichment. However, we consider this unlikely as we did not observe any MS^2^ scans for MAMI when we performed the enrichment with the Af1521 G42E variant (Dani et al. 2009) that does not bind ADPr (0 MS^2^ scans G42E vs. 15 MS^2^ scans Af1521; Table 1 and Dataset S4). We obtained further evidence for AvrRpm1‐mediated ADP‐ribosylation of MAMI from the transient *N. benthamiana* expression system. When AvrRpm1‐Flag was coexpressed with a HA‐tagged MAMI, we observed an additional slower migrating band on the α‐HA immunoblot (Figures 3B and S3). A band of ~37 kDa was also visible when the blot was probed with α‐pan‐ADPr (Figure 3B), indicating that MAMI can be ADP‐ribosylated by AvrRpm1. In LC–MS/MS analysis of samples from *N. benthamiana* leaves coexpressing AvrRpm1‐Flag and HA‐MAMI, we detected the ADP‐ribosylated MAMI peptide shown in Figure 3A only in the pulldown with the functional Af1521 Macro domain but not the G42E control. Expression of HA‐MAMI alone did not result in detectable ADP‐ribosylation indicating that coexpression of AvrRpm1 was required for ADP‐ribosylation of MAMI (Dataset S5).

We searched Dataset S4 for additional proteins that were specifically enriched by the functional versus nonfunctional Af1521 Macro domain. Overall, the dataset contains 559 protein groups identified by at least two MS^2^ scans. The overlap was 87% with 77 protein groups only identified in the Af1521 samples, whereas six protein groups were unique to the G42E pulldowns (Dataset S4). A subset of 65 protein groups was detected in all three experiments of at least one dataset and was compared by LFQ in MaxQuant. Apart from the Af1521 Macro bait protein, the following four proteins were significantly enriched in the pulldown with the Af1521 Macro domain: MAMI, the blue light photoreceptor PHOTOTROPIN 1 (PHOT1, AT3G45780.2), PHOSPHOLIPASE D GAMMA 3 (PLDGAMMA3, AT4G11840.2), and a PapD‐like superfamily protein (AT4G21450.1) (Table 1, Figure S4, and Dataset S1 and S4). Therefore, even if no ADP‐ribosylated peptides can be identified, comparative pulldowns with the functional versus nonfunctional Af1521 Macro domains can provide information on potentially modified proteins that can be further assessed for evidence of ADP‐ribosylation by directed methods. We attempted to identify ADP‐ribosylated peptides of PHOT1 and PLDGAMMA3 by coexpressing these proteins with AvrRpm1‐Flag in *N. benthamiana*, followed by affinity enrichment of ADP‐ribosylated proteins and detection by LC–MS/MS. While we did not identify any ADP‐ribosylated peptides from PHOT1, profiling of samples coexpressing PLDGAMMA3‐HA and AvrRpm1‐Flag revealed the N‐terminal acetylated peptide shown in Figure 3C from the phospholipase with Q23 as the most likely ADP‐ribosylation site in two out of three replicates (Dataset S6, see Figure S5A for an annotated MS^2^ spectrum). Notably, in Dataset S6, we also detected ADP‐ribosylated peptides from *N. benthamiana* RIN4 homologs (NbD024504.1, NbE03056010.1, NbD049321.1, and NbE03061280.1) and from proteins with sequence homology to Arabidopsis MAMI (NbD046047.1, NbE03057442.1, NbD027840.1, and NbD012133.1). This observation further strengthens the identification of these proteins as substrates of AvrRpm1.

To assess if the ADPr profiling method is applicable to other host‐targeted ADP‐ribosyltransferases from *Pst*, we extended our analysis to transgenic Arabidopsis lines that conditionally express the T3E HopU1. HopU1 ADP‐ribosylates the RNA‐binding protein GRP7 and two Arginine residues (R47 and R49) are essential for HopU1‐mediated ADP‐ribosylation (Fu et al. 2007). ADP‐ribosylation on R49, a residue with an important role in RNA binding (Schöning et al. 2007), has been confirmed by in vitro enzyme assays with recombinantly expressed proteins followed by proteomics (Jeong et al. 2011). Dex‐induced expression of HopU1‐Flag resulted in three prominent bands detected on an α‐pan‐ADPr immunoblot (Figure S5). The band migrating just above the 15‐kDa marker would be consistent with the molecular weight of GRP7 (16 kDa). We applied the ADPr profiling method to plants expressing HopU1‐Flag and compared them to Col‐0 plants. We identified the ADP‐ribosylated GRP7 peptide SRGFGFVTFKDEK (48–60) with a possible modification on S48 or R49 in two replicates (Figure S5). The modified peptide was specifically detected in the Af1521‐enriched samples and not in the G42E controls or Col‐0 samples (Dataset S7). We conclude that the ADPr profiling method is applicable to different T3Es as long as they induce sufficient levels of plant protein ADP‐ribosylation when expressed as a transgene (Figure S5).

## 4 Discussion

In this study, we present a proteomics workflow that, regardless of prior knowledge, would have identified RIN4 and sequence‐related NOI proteins as substrates of the host‐targeted bacterial ADP‐ribosyltransferase AvrRpm1 in Arabidopsis. The method identified the two ADP‐ribosylation sites on the endogenous RIN4 protein that were predicted by Redditt et al. (2019) based on LC–MS/MS analysis of the soybean RIN4b protein. We further show that RIN4 homologs NOI4 and NOI12 are ADP‐ribosylated at the sequence‐equivalent site of the conserved NOI sequence, indicating that AvrRpm1 preferentially modifies NOI proteins on Glu, Asp, or Asn residues at the fifth position of the PKFG[E/D/N]W[E/D/N] consensus sequence. AvrRpm1 and the sequence‐unrelated T3E AvrB indirectly promote RIN4 phosphorylation on T166 by receptor‐like cytoplasmic kinases, and it is the AvrRpm1‐mediated manipulation of the RIN4 C‐NOI sequence that is sensed by RPM1 (Liu et al. 2011; Chung et al. 2011).

In the absence of NLR recognition, that is, when AvrRpm1‐expressing *P. syringae* bacteria are directly infiltrated into leaves of the *rpm1 rps2* double mutant, AvrRpm1 promotes bacterial virulence, and this does not require RIN4 (Belkhadir et al. 2004). One possible explanation would be potential functional redundancy among the RIN4/NOI protein family members. A not mutually exclusive scenario is that plasma membrane‐associated proteins like MAMI, PLDGAMMA3, or PHOT1, that are specifically ADPr‐enriched from AvrRpm1‐expressing plants, might constitute potential virulence targets of AvrRpm1. The PHOT1 orthologs of potato and the liverwort *Marchantia polymorpha* have recently been identified as negative regulators of immunity, and the potato late blight pathogen *Phytophthora infestans* employs an effector protein to manipulate immune signaling downstream of Stphot1 (He et al. 2018; Naqvi et al. 2022; Yotsui et al. 2023). These findings may support a role of Arabidopsis PHOT1 as a virulence target of AvrRpm1. However, given that the Dex‐induced AvrRpm1 effector is overexpressed in our experimental system, this hypothesis needs to be tested by bacterial growth assays in a *rpm1 rps2 phot1* triple mutant background.

In contrast to AvrRpm1 and HopU1, our approach did not identify substrates of HopF2. Multiple host targets of HopF2 have been proposed, including MKK5, RIN4, BAK1, and QSK1 (Y. Wang et al. 2010; Wilton et al. 2010; Zhou et al. 2014; Goto et al. 2024). However, none of these proteins were enriched in ADPr affinity pulldowns from HopF2‐expressing plant cell extracts (Dataset S1). One possible explanation is that proteins targeted by HopF2 have low abundance, are not effectively extracted by our protein isolation method, and/or are rapidly degraded upon modification. However, at least for the proposed HopF2 target RIN4, we can exclude inappropriate extraction conditions or low abundance, as proven by the successful affinity enrichment of ADP‐ribosylated RIN4 from transgenic lines expressing AvrRpm1. The Af1521 Macro domain has been used for proteome‐wide profiling of ADP‐ribosylation sites in mammalian cells and does not show strong preferences in terms of the modified amino acid (Martello et al. 2016; Hendriks et al. 2019), although a recent publication found a preference for arginine‐ADPr (Tashiro et al. 2023). Y. Wang et al. (2010) reported that a constitutively active MKK5 variant is more efficiently ADP‐ribosylated by HopF2, suggesting that the kinase might require prior activation for effector‐induced modification. At least in vitro, we were able to reproduce the HopF2‐dependent ADP‐ribosylation of MKK5 by using an α‐ADPr antibody (Figure S6). We consider the differential efficiency of MKK5 ADP‐ribosylation in planta and in vitro a possible reason for the lack of ADPr peptide enrichment from plants expressing HopF2.

We envisage that the method presented here will facilitate untargeted proteomics approaches to identify substrates of ADP‐ribosyltransferases in plants. LC–MS/MS profiling of proteins that are enriched by the Af1521 Macro domain but not the G42E mutant can provide information on plant proteins that are ADP‐ribosylated by a particular effector by comparison to suitable controls. The “triggering” proteomics method has the potential to directly sequence modified ADPr‐enriched peptides as demonstrated for NOI4, NOI12, RIN4, MAMI, and GRP7. As shown for MAMI and PLDGAMMA3, the transient *N. benthamiana* expression system can aid in pinpointing the ADP‐ribosylated peptide and/or amino acid. Ideally, an efficient and specific enrichment by the Af1521 Macro domain, paired with detection of ADP‐ribosylated peptides, will facilitate the identification of proteins modified by host‐targeted ADP‐ribosyltransferases from pathogens.

In contrast to the successful identification of a number of ADP‐ribosylating effector substrates, our method did not enable reliable detection of plant proteins that are ADP‐ribosylated by endogenous plant ADP‐ribosyltransferases. Other studies, for example, on mammalian cell cultures under oxidative stress, reported hundreds of ADP‐ribosylated peptides (Martello et al. 2016; Hendriks et al. 2019). The corresponding methods are based on cell lysis under denaturing conditions, enrich ADP‐ribosylated peptides and not proteins, and rely on incubation of peptides with poly(ADPr)glycohydrolases (PARGs) that hydrolyze poly(ADPr) chains to mono‐ADPr. We did not include an incubation with recombinant PARG enzyme in our approach as there is currently no evidence that *P. syringae* T3Es catalyze poly(ADP‐ribosylation). Furthermore, several examples of PARG‐catalyzed hydrolysis of mono‐ADP‐ribosylation in vitro and in cells have been reported, indicating that an incubation step with excess PARG enzyme could result in loss of ADP‐ribosylation sites (Kong et al. 2021; Tashiro et al. 2023; Rack et al. 2024; Longarini and Matić 2024). Apart from differences in the experimental approach, other possible reasons for the lack of ADP‐ribosylation sites set by endogenous plant “writer” enzymes in our analysis could be a lower abundance compared to the ADP‐ribosylation reactions catalyzed by overexpressed pathogen effectors.

### 4.1 Accession Numbers

AvrRpm1 Q7BE94, HopU1 Q88A91, HopF2 Q88A90, RIN4 AT3G25070, NOI4 AT5G55850, NOI12 AT2G04410, MAMI AT5G54110, PLDGAMMA3 AT4G11840, PHOT1 AT3G45780, GRP7 AT2G21660, PERK1 AT3G24550, PIN3 AT1G70940, TLD‐domain containing nucleolar protein AT5G39590, SIT4 phosphatase‐associated family protein AT1G30470, TAP46 AT5G53000, and pOPINF Addgene (https://www.addgene.org/) plasmid #26042.

## Author Contributions

S.K., T.C., and L.W. conceived and designed experiments. S.K., D.T., C.P., and L.W. carried out experiments. S.K., T.C., S.M., I.M., and L.W. analyzed the data. L.W. wrote the manuscript with input from all co‐authors. All authors reviewed and approved the submitted manuscript.

## Conflicts of Interest

The authors declare no conflicts of interest.

## Peer Review

The peer review history for this article is available in the Supporting Information for this article.

## Supporting information




**Data S1:** Peer Review.

**Data S2:** Supplementary methods.

**Dataset S1:** Proteins identified by untargeted proteomics in four Af1521 Macro pulldown experiments from Dex‐treated Col‐0, AvrRpm1‐HA, and HopF2‐HA plants.

**Datasets S2 and S3:** ADP‐ribosylated peptides identified in three Af1521 Macro pulldown experiments from AvrRpm1‐HA samples by the standard method (S2) and the “triggering” method (S3).

**Dataset S4:** Comparison of proteins identified in three experiments using the functional (Af1521) versus nonfunctional (G42E) Macro domains following ADPr‐enrichment of proteins from Dex‐treated AvrRpm1‐HA plants.

**Dataset S5:** AvrRpm1 ADP‐ribosylates MAMI when expressed in *Nicotiana benthamiana*.

**Dataset S6:** AvrRpm1 ADP‐ribosylates PLDGAMMA3 when expressed in *Nicotiana benthamiana*.

**Dataset S7:** ADP‐ribosylated peptides identified in two Af1521 Macro pulldown experiments from HopU1‐Flag versus Col‐0 samples by the “triggering” method.

**Dataset S8:** Detailed description of proteomics methods used in this work.

**Figure S1:** Comparison of proteins enriched by the Af1521 Macro domain from Col‐0, AvrRpm1‐, and HopF2‐expressing lines based on LFQ. Volcano plots with FDR 0.05 show significant differences between Col‐0 and AvrRpm1‐expressing samples (A) and no significant differences between Col‐0 and HopF2‐expressing samples (B).

**Figure S2:** (A) Volcano plot with FDR 0.05 showing significant differences between proteins identified in Datasets S2 and S3. (B) Scatter plots showing pairwise comparisons of all samples from Datasets S2 and S3 based on LFQ values. The respective Pearson correlations are indicated in the individual plots. (C) Radar plots comparing quality of the spectra from ADP‐ribosylated peptides between the standard method (Dataset S2) and the “triggering” method (Dataset S3). “# proteins” = number of identified ADP‐ribosylated proteins. “ADPr sites” = number of identified ADP‐ribosylation sites on peptides. “score” = average Andromeda score of the ADP‐ribosylated peptides. “loc. score” = average localization score for ADP‐ribosylation. “loc. probability”= average localization probability for ADP‐ribosylation. “% ADPr marker” = % of spectra with ADPr marker ions. “% loc. prob. > 0.99” = % of spectra with a localization probability of > 0.99 for ADP‐ribosylation.

**Figure S3:** Images of the full membranes used for Figure 3B.

**Figure S4:** Volcano plot with FDR 0.05 showing significant differences between proteins enriched by the functional versus nonfunctional Af1521 Macro domain from AvrRpm1‐expressing plants.

**Figure S5:** (A) HCD MS^2^ spectrum of the ADP‐ribosylated peptide AYHPVYNETMSMGGGSSNEFGQWLDK (*z* = 3, mass = 3519.31 Da) from PLDGAMMA3‐HA‐StrepII coexpressed with AvrRpm1‐Flag in *Nicotiana benthamiana*. (B) HCD MS^2^ spectrum of the ADP‐ribosylated GRP7 peptide SRGFGFVTFKDEK (*z* = 3, mass = 2057.83 Da) identified from HopU1‐expressing Arabidopsis plants. The ADPr marker ions are shown in beige. Unmodified fragment ions and fragment ions with a neutral loss of AMP (Δ347.06 Da) are indicated in the sequence logos. The horizontal line designates the possible sequence window for ADP‐ribosylation. (C) Inducible expression of the T3E HopU1 results in ADP‐ribosylation of proteins in Arabidopsis. Protein extracts from Dex‐treated Col‐0 wild type and the indicated transgenic lines were separated by SDS‐PAGE, electroblotted onto PVDF membrane, and probed with α‐HA, a‐Flag, or α‐pan‐ADPr antibodies. The Amido black–stained membrane is presented as loading control.

**Figure S6:** HopF2 can ADP‐ribosylate MKK5 in vitro. His6‐MKK5 or the control protein His6‐HaRxL106ΔC were incubated with His6‐HopF2 in ADP‐ribosylation buffer at 25°C for 45 min. In control reactions, the equivalent volume of buffer was added instead of His6‐HopF2. ADP‐ribosylation was detected by immunoblotting with α‐ADPr E6F6A antibody. As loading control, the same volume of the samples was analyzed by SDS‐PAGE.

**Table S1:** Comparison of ADP‐ribosylation sites identified in Datasets S2 (standard method) and S3 (triggering method).

**Table S2:** Comparison of ADP‐ribosylation sites identified with 1, 2.5, and 5 μg of Af1521 Macro domain in Datasets S2 and S3.

## Data Availability

The following plasmids from this work have been submitted to Addgene: pENTR4‐MAMI #209186, pENTR4‐PHOT1 #209187, pENTR4‐PLDGAMMA3 #209188, pENTR4‐AvrRpm1 #209185, pENTR4‐HopU1 #228395, pOPINF‐HopF2 #228397, and pOPINF‐MKK5 #228396. Sequence and structure of the enhanced Af1521 Macro domain has been published by Nowak et al. (2020) at https://www.rcsb.org/structure/6FX7. The pOPINF His6‐GST‐Af1521 Macro expression constructs are in part bound by a MTA with the University of Zurich and are, as all other materials, available upon request. The mass spectrometry proteomics data have been submitted to the ProteomeXchange Consortium via the PRIDE (Perez‐Riverol et al. 2022) partner repository with the following identifiers: PXD068442 (Dataset S1), PXD068665 (Dataset S2 and S3), PXD068551 (Dataset S4), PXD068485 (Dataset S5), PXD068675 (Dataset S6), and PXD068739 (Dataset S7).
