# Supplementary material for: Untargeted Proteomics Identifies Plant Substrates of the Bacterial‐Derived ADP‐Ribosyltransferase AvrRpm1
Source: Plant Direct. 2025 Nov 16;9(11):e70115. doi: 10.1002/pld3.70115 (PMC12620056; doi:10.1002/pld3.70115)
Supplement: Supplementary file 2 — Data S2: Supplementary methods. [file PLD3-9-e70115-s003.docx]

**Supplementary methods**

Protein expression and purification

His6-GST-Macro variants, His6-HopF2, and His6-MKK5 were expressed in *E. coli* Shuffle cells (NEB). Cultures were grown in LB medium at a temperature of 37 °C to an OD_600_ of 1.0 – 1.2. The cultures were cooled to 18 °C before expression was induced by the addition of 0.5 mM IPTG for 16 h. Cells were pelleted by centrifugation (5000 x *g* / 4 °C / 12 min) and the pellets were resuspended in buffer A [50 mM Tris-HCl, 0.3 M NaCl, 20 mM imidazole, 5% (v/v) glycerol, 50 mM glycine, pH 8.0] supplemented with 0.1% polyethylenimine and 1x cOmplete™ EDTA-free protease inhibitor cocktail (Merck). Cells lysis was induced by addition of Lysozyme (1 mg/mL final concentration / 25 °C / 15 min) followed by sonication on ice (Branson 150D Sonifier, 2x 10 min, level 3-4). Insoluble proteins and cell debris were removed by centrifugation (30000 x *g* / 4 °C / 30 min) and the supernatant was loaded onto a 5 mL HisTrap HP IMAC column (Cytiva). The column was washed with buffer A until the A_280_ reached 25 mAU and proteins were eluted using buffer B [50 mM Tris-HCl, 0.3 M NaCl, 0.5 M imidazole, 5% (v/v) glycerol, 50 mM glycine, pH 8.0]. The elution from the IMAC column was injected onto a size exclusion chromatography column [Superdex 75 26/60 PG column (Cytiva) pre-equilibrated with 20 mM HEPES-NaOH, 150 mM NaC, pH 7.5]. Proteins eluting from the column were concentrated by ultrafiltration on Vivaspin 20 columns (Sartorius) with a 5 kDa molecular weight cut-off. For His6-MKK5 we selected the peak corresponding to the monomeric form. For the His6-GST-Macro variants the tag was cleaved using 3C protease. The protein was run through a 5 mL HisTrap HP IMAC column in buffer A to remove the His6-tag and residual un-cleaved fusion protein, followed by injection onto the Superdex 75 26/60 PG column and elution and concentration as above. Aliquots were snap-frozen in liquid N_2_ and stored at -70 °C

*In vitro* ADP-ribosylation assay

8 μg of His6-MKK5 [or His6-HaRxL106ΔC as control protein (Wirthmueller et al., 2015)] was diluted in reaction buffer (40 mM HEPES pH7.5, 5 mM MgCl_2_, 1 mM DTT, 30 μM NAD^+^, 60 μM ATP). Then either 4 μg His6-HopF2 or the corresponding volume of buffer (20 mM HEPES pH7.5, 150 mM NaCl) were added to a final volume of 50 μL. The reactions were incubated at 25 °C for 45 min. The samples were supplemented with 17 μL 4x SDS sample buffer and incubated at 75 °C for 3 min. ADP-ribosylation was detected by an immunoblot with α-ADPr (E6F6A) antibody as described above. As control, proteins were separated by SDS-PAGE and visualized by staining with Instant Blue (Abcam).

Reference

Wirthmueller, L., Roth, C., Fabro, G., Caillaud, M.-C., Rallapalli, G., Asai, S., Sklenar, J., Jones, A.M.E., Wiermer, M., Jones, J.D.G., and Banfield, M.J. (2015). Probing formation of cargo/importin-α transport complexes in plant cells using a pathogen effector. Plant J. 81: 40–52.
