# Supplementary material for: Untargeted Proteomics Identifies Plant Substrates of the Bacterial‐Derived ADP‐Ribosyltransferase AvrRpm1
Source: Plant Direct. 2025 Nov 16;9(11):e70115. doi: 10.1002/pld3.70115 (PMC12620056; doi:10.1002/pld3.70115)
Supplement: Supplementary file 10 — Figure S1: Comparison of proteins enriched by the Af1521 Macro domain from Col‐0, AvrRpm1‐, and HopF2‐expressing lines based on LFQ. Volcano plots with FDR 0.05 show significant differences between Col‐0 and AvrRpm1‐expressing samples (A) and no significant differences between Col‐0 and HopF2‐expressing samples (B). [file PLD3-9-e70115-s006.pdf]

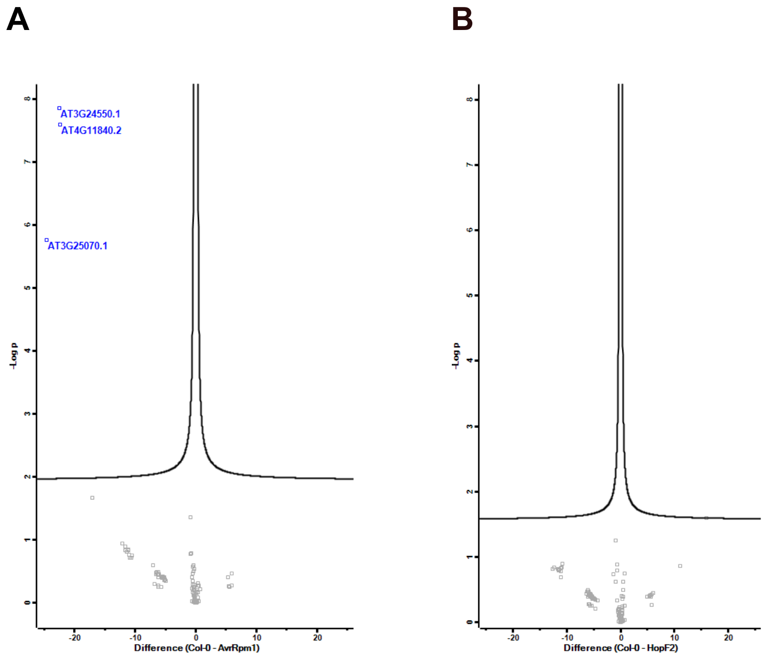

Supplementary Figure S1. Comparison of proteins enriched by the Af1521 Macro domain from Col-0, AvrRpm1-, and HopF2-expressing lines based on LFQ. Volcano plots with FDR 0.05 show significant differences between Col-0 and AvrRpm1-expressing samples (**A**) and no significant differences between Col-0 and HopF2-expressing samples (**B**).
