# Supplementary material for: Untargeted Proteomics Identifies Plant Substrates of the Bacterial‐Derived ADP‐Ribosyltransferase AvrRpm1
Source: Plant Direct. 2025 Nov 16;9(11):e70115. doi: 10.1002/pld3.70115 (PMC12620056; doi:10.1002/pld3.70115)
Supplement: Supplementary file 11 — Figure S2: (A) Volcano plot with FDR 0.05 showing significant differences between proteins identified in Datasets S2 and S3. (B) Scatter plots showing pairwise comparisons of all samples from Datasets S2 and S3 based on LFQ values. The respective Pearson correlations are indicated in the individual plots. (C) Radar plots comparing quality of the spectra from ADP‐ribosylated peptides between the standard method (Dataset S2) and the “triggering” method (Dataset S3). “# proteins” = number of identified ADP‐ribosylated proteins. “ADPr sites” = number of identified ADP‐ribosylation sites on peptides. “score” = average Andromeda score of the ADP‐ribosylated peptides. “loc. score” = average localization score for ADP‐ribosylation. “loc. probability”= average localization probability for ADP‐ribosylation. “% ADPr marker” = % of spectra with ADPr marker ions. “% loc. prob. > 0.99” = % of spectra with a localization probability of > 0.99 for ADP‐ribosylation. [file PLD3-9-e70115-s012.pdf]

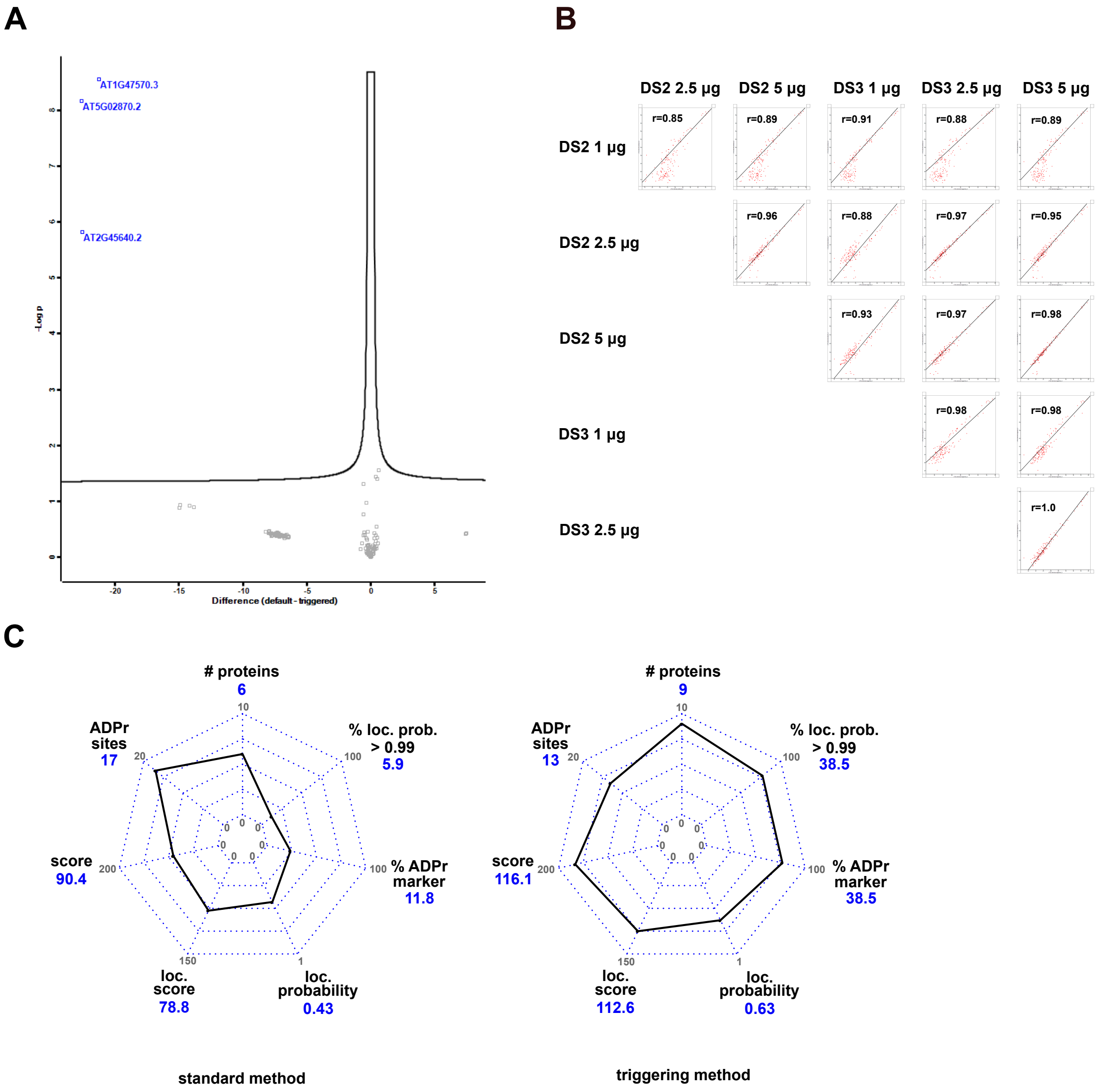

Supplementary Figure S2. **(A)** Volcano plot with FDR 0.05 showing significant differences between proteins identified in Supplementary Datasets S2 and S3. **(B)** Scatter plots showing pairwise comparisons of all samples from Supplementary Datasets S2 and S3 based on LFQ values. The respective Pearson correlations are indicated in the individual plots. **(C)** Radar plots comparing quality of the spectra from ADP-ribosylated peptides between the standard method (Supplementary Dataset S2) and the 'triggering' method (Supplementary Dataset S3). '# proteins' = number of identified ADP-ribosylated proteins. 'ADPr sites' = number of identified ADP-ribosylation sites on peptides. 'score' = average Andromeda score of the ADP-ribosylated peptides. 'loc. score' = average localization score for ADP-ribosylation. 'loc. probability' = average localization probability for ADP-ribosylation. '% ADPr marker' = % of spectra with ADPr marker ions. '% loc. prob. >0.99' = % of spectra with a localization probability of >0.99 for ADP-ribosylation.
