# Supplementary figures and images for: Untargeted Proteomics Identifies Plant Substrates of the Bacterial‐Derived ADP‐Ribosyltransferase AvrRpm1
Source: Plant Direct. 2025 Nov 16;9(11):e70115. doi: 10.1002/pld3.70115 (PMC12620056; doi:10.1002/pld3.70115)

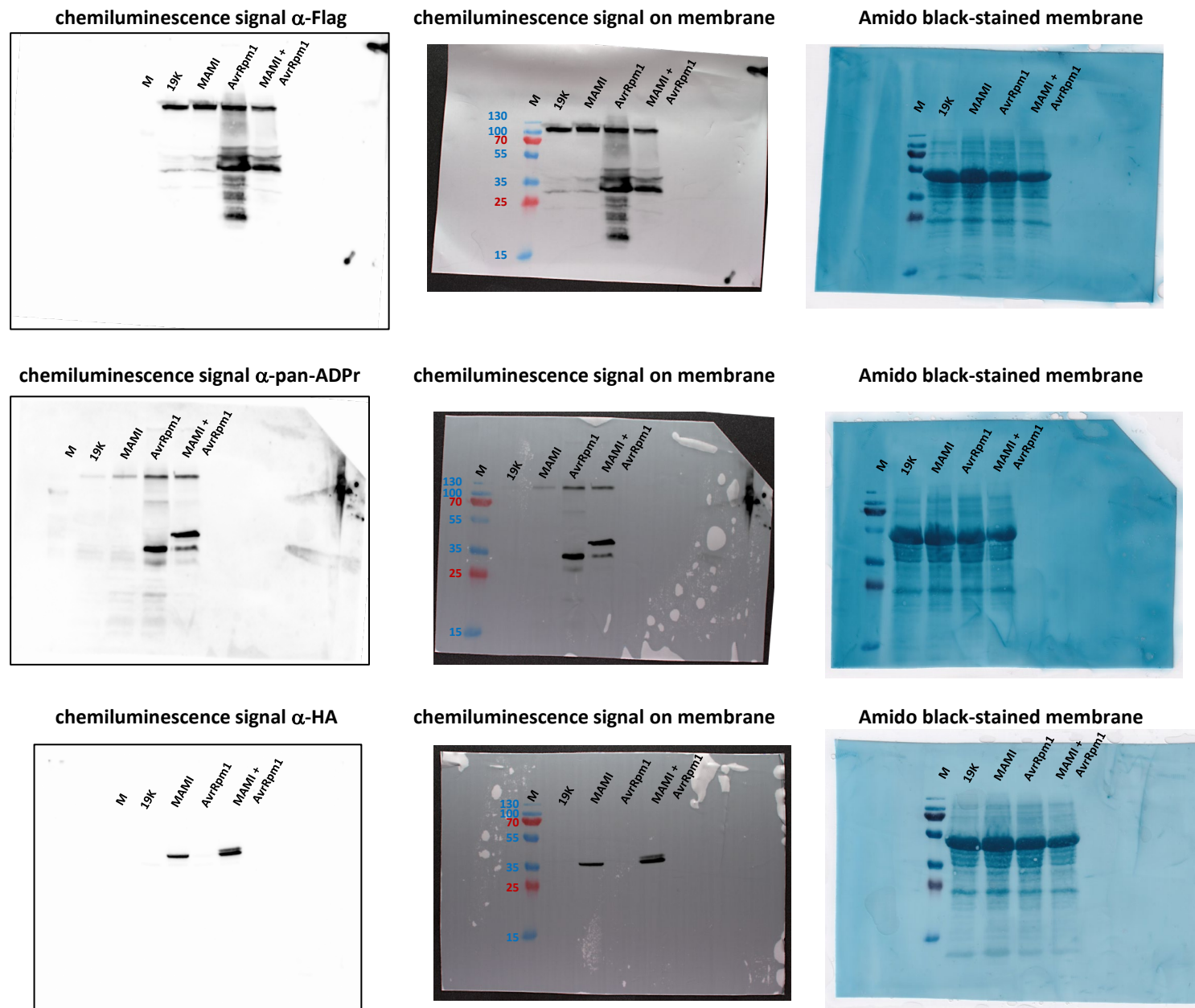

Supplementary Figure S3. Images of the full membranes used for Figure 3B.

Supplement: Supplementary file 12 — Figure S3: Images of the full membranes used for Figure 3B. [file PLD3-9-e70115-s016.pdf]
