# Supplementary material for: Untargeted Proteomics Identifies Plant Substrates of the Bacterial‐Derived ADP‐Ribosyltransferase AvrRpm1
Source: Plant Direct. 2025 Nov 16;9(11):e70115. doi: 10.1002/pld3.70115 (PMC12620056; doi:10.1002/pld3.70115)
Supplement: Supplementary file 13 — Figure S4: Volcano plot with FDR 0.05 showing significant differences between proteins enriched by the functional versus nonfunctional Af1521 Macro domain from AvrRpm1‐expressing plants. [file PLD3-9-e70115-s014.pdf]

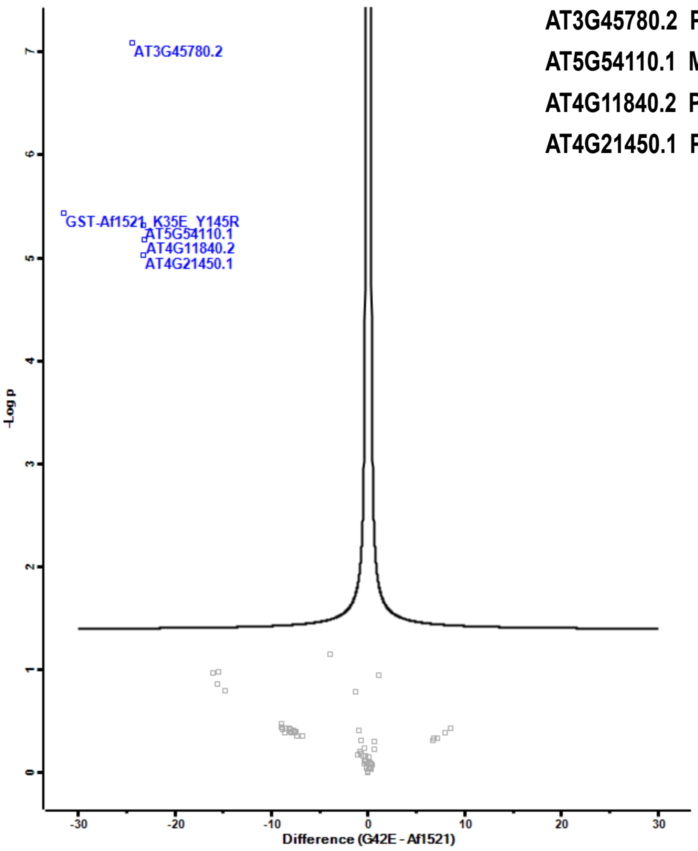

AT3G45780.2 PHOT1

AT5G54110.1 MAMI

AT4G11840.2 PLDGAMMA3

AT4G21450.1 PapD-like superfamily protein

Supplementary Figure S4. Volcano plot with FDR 0.05 showing significant differences between proteins enriched by the functional vs. non-functional Af1521 Macro domain from AvrRpm1-expressing plants.
