# Supplementary material for: Untargeted Proteomics Identifies Plant Substrates of the Bacterial‐Derived ADP‐Ribosyltransferase AvrRpm1
Source: Plant Direct. 2025 Nov 16;9(11):e70115. doi: 10.1002/pld3.70115 (PMC12620056; doi:10.1002/pld3.70115)
Supplement: Supplementary file 14 — Figure S5: (A) HCD MS2 spectrum of the ADP‐ribosylated peptide AYHPVYNETMSMGGGSSNEFGQWLDK (z = 3, mass = 3519.31 Da) from PLDGAMMA3‐HA‐StrepII coexpressed with AvrRpm1‐Flag in Nicotiana benthamiana. (B) HCD MS2 spectrum of the ADP‐ribosylated GRP7 peptide SRGFGFVTFKDEK (z = 3, mass = 2057.83 Da) identified from HopU1‐expressing Arabidopsis plants. The ADPr marker ions are shown in beige. Unmodified fragment ions and fragment ions with a neutral loss of AMP (Δ347.06 Da) are indicated in the sequence logos. The horizontal line designates the possible sequence window for ADP‐ribosylation. (C) Inducible expression of the T3E HopU1 results in ADP‐ribosylation of proteins in Arabidopsis. Protein extracts from Dex‐treated Col‐0 wild type and the indicated transgenic lines were separated by SDS‐PAGE, electroblotted onto PVDF membrane, and probed with α‐HA, a‐Flag, or α‐pan‐ADPr antibodies. The Amido black–stained membrane is presented as loading control. [file PLD3-9-e70115-s007.pdf]

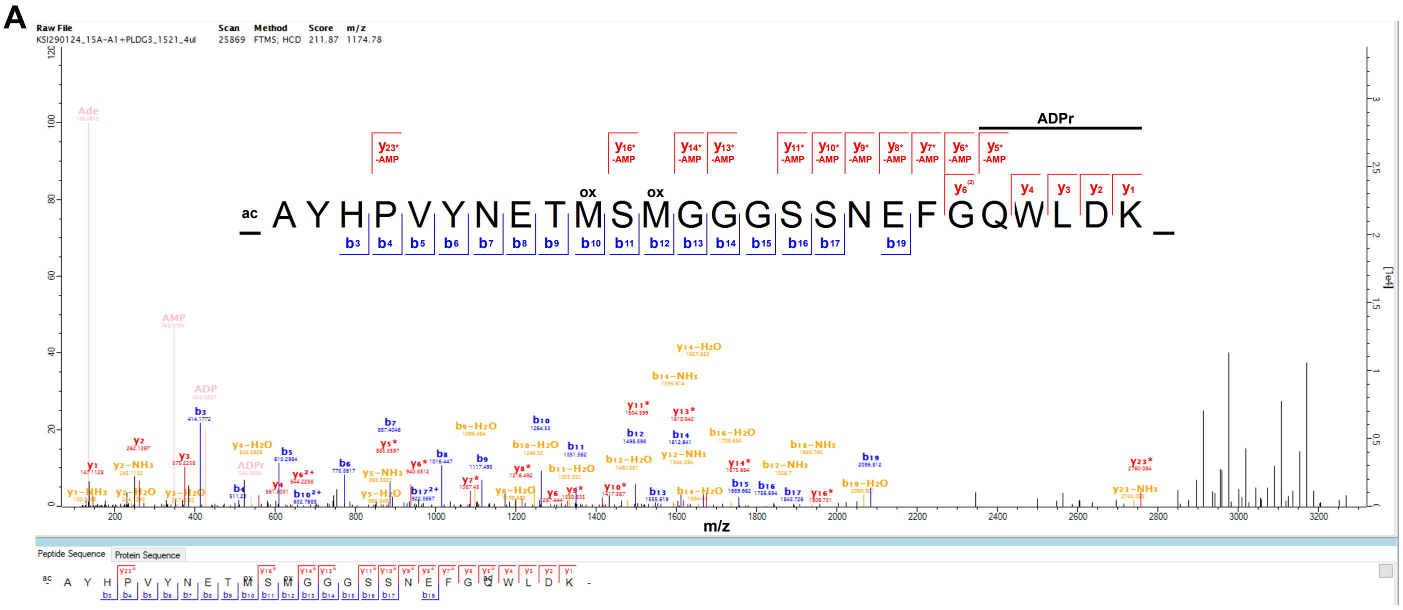

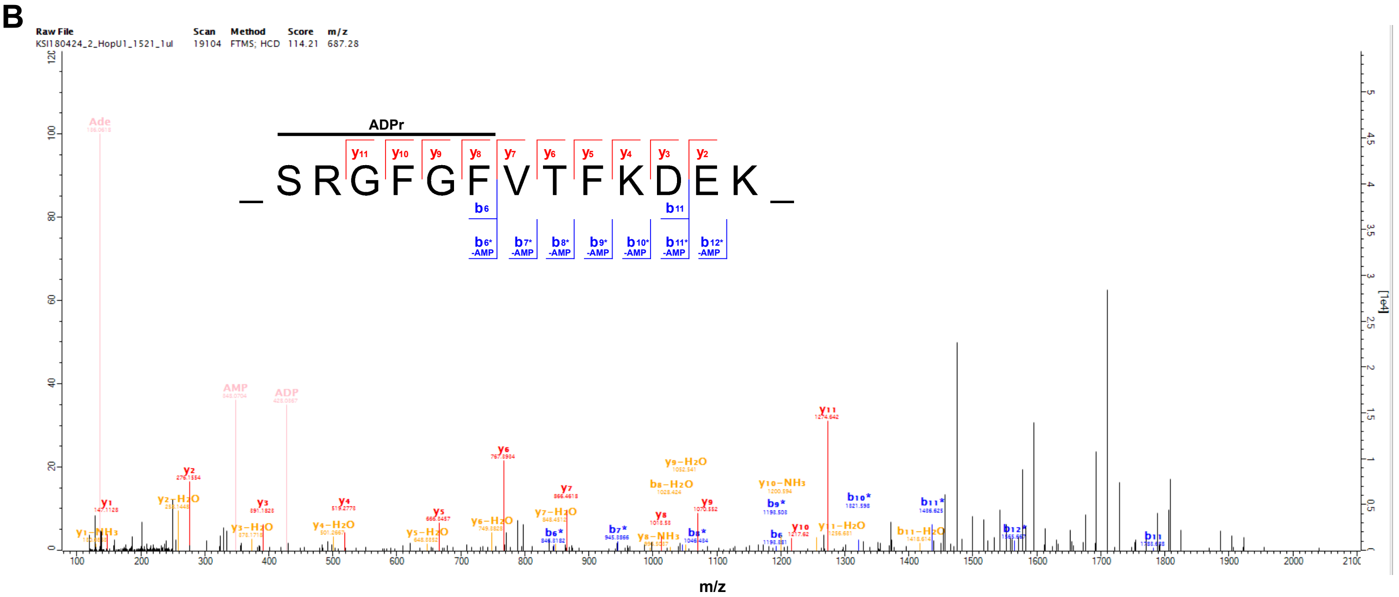

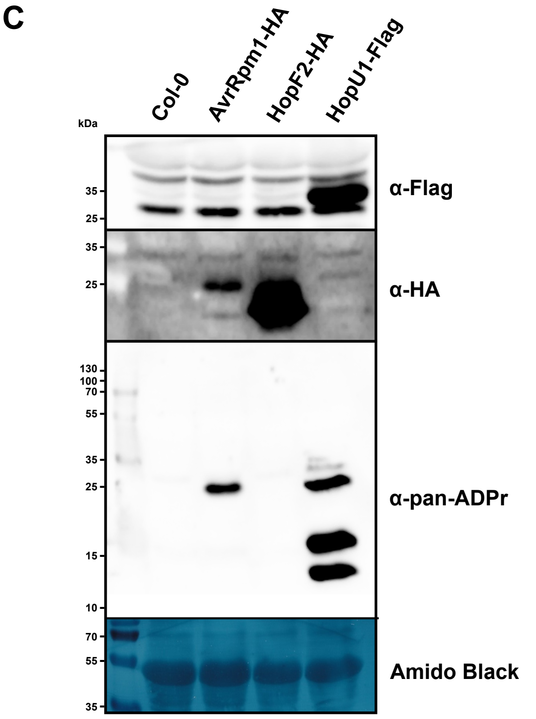

Supplementary Figure S5. **(A)** HCD MS^2 spectrum of the ADP-ribosylated peptide AYHPVYNETMSMGGGSSNEFGQWLDK (z = 3, mass = 3519.31 Da) from PLDGAMMA3-HA-StrepII co-expressed with AvrRpm1-Flag in *N. benthamiana*. **(B)** HCD MS^2 spectrum of the ADP-ribosylated GRP7 peptide SRGFGFVTFKDEK (z = 3, mass = 2057.83 Da) identified from HopU1-expressing Arabidopsis plants. The ADPr marker ions are shown in beige. Unmodified fragment ions and fragment ions with a neutral loss of AMP (Δ347.06 Da) are indicated in the sequence logos. The horizontal line designates the possible sequence window for ADP-ribosylation. **(C)** Inducible expression of the T3E HopU1 results in ADP-ribosylation of proteins in Arabidopsis. Protein extracts from Dex-treated Col-0 wild type and the indicated transgenic lines were separated by SDS-PAGE, electroblotted onto PVDF membrane and probed with α-HA, a-Flag or α-pan-ADPr antibodies. The Amido Black-stained membrane is presented as loading control.
