# Supplementary material for: Untargeted Proteomics Identifies Plant Substrates of the Bacterial‐Derived ADP‐Ribosyltransferase AvrRpm1
Source: Plant Direct. 2025 Nov 16;9(11):e70115. doi: 10.1002/pld3.70115 (PMC12620056; doi:10.1002/pld3.70115)
Supplement: Supplementary file 15 — Figure S6: HopF2 can ADP‐ribosylate MKK5 in vitro. His6‐MKK5 or the control protein His6‐HaRxL106ΔC were incubated with His6‐HopF2 in ADP‐ribosylation buffer at 25°C for 45 min. In control reactions, the equivalent volume of buffer was added instead of His6‐HopF2. ADP‐ribosylation was detected by immunoblotting with α‐ADPr E6F6A antibody. As loading control, the same volume of the samples was analyzed by SDS‐PAGE. [file PLD3-9-e70115-s005.pdf]

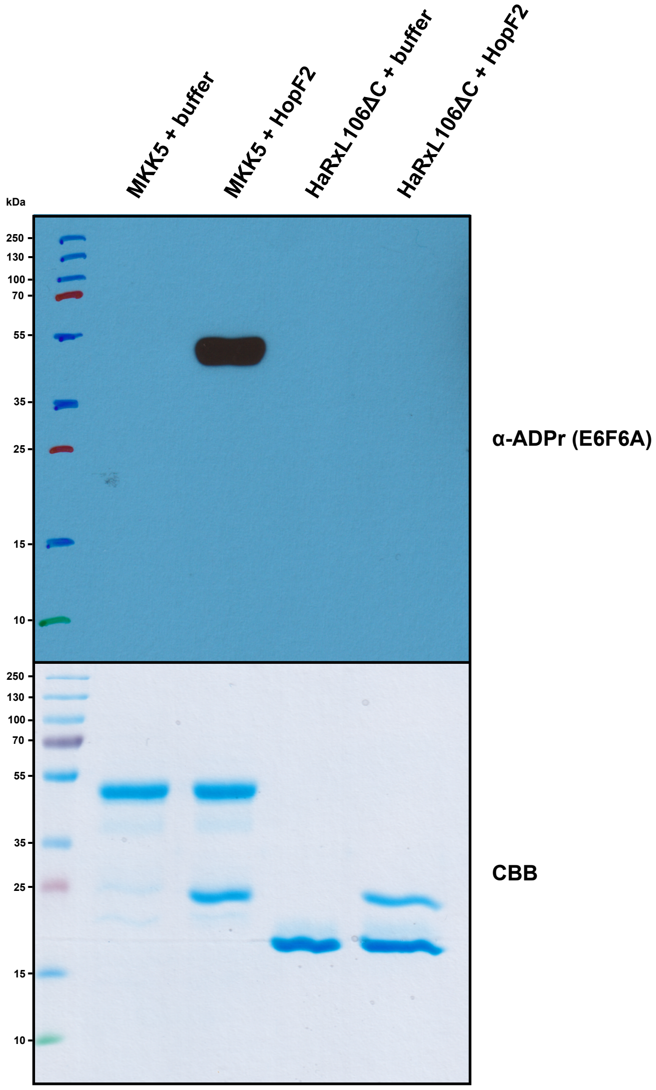

Supplementary Figure S6. HopF2 can ADP-ribosylate MKK5 *in vitro*. His6-MKK5 or the control protein His6-HaRxL106ΔC were incubated with His6-HopF2 in ADP-ribosylation buffer at 25 °C for 45 min. In control reactions the equivalent volume of buffer was added instead of His6-HopF2. ADP-ribosylation was detected by immunoblotting with α-ADPr E6F6A antibody. As loading control, the same volume of the samples was analyzed by SDS-PAGE.
