# Supplementary material for: Untargeted Proteomics Identifies Plant Substrates of the Bacterial‐Derived ADP‐Ribosyltransferase AvrRpm1
Source: Plant Direct. 2025 Nov 16;9(11):e70115. doi: 10.1002/pld3.70115 (PMC12620056; doi:10.1002/pld3.70115)
Supplement: Supplementary file 1 — Data S1: Peer Review. [file PLD3-9-e70115-s002.pdf]

Plant Direct
Open Access

Untargeted proteomics identifies plant substrates of the bacterial-derived ADP-ribosyltransferase AvrRpm1

Dr. Lennart Wirthmueller, Ms. Simranjit Kaur , Dr. Thomas Colby , Ms. Domenika Thieme , Mr. Carsten Proksch , Dr. Susanne Matschi , Dr. Ivan Matić

Decision Letter Round 1:

January 7, 2025

Dr. Lennart Wirthmueller

Leibniz-Institut fur Pflanzenbiochemie

Biochemistry of Plant Interactions

Weinberg 3

06120

Halle 14195

Germany

RE: Untargeted proteomics identifies plant substrates of the bacterial-derived ADP-ribosyltransferase AvrRpm1

Dear Dr. Wirthmueller:

Thank you for submitting to Plant Direct. All required reviews have been returned and we have now finished our evaluation of your manuscript. In light of the reviewers' and editor's comments, further revisions are needed before the paper can be accepted for publication in Plant Direct.

Please view the editors' and reviewers' comments below and use their suggestions as a guide while you work on your revision.

When uploading the revised version of this article, please be sure to include the following:

-A word document that contains your response to the reviewers. You should respond to each reviewer comment and note the changes made to the manuscript. If you do not agree with a reviewer's comment and choose not to make a suggested revision, please explain why. Please try to provide as complete an answer as possible to each reviewer's criticisms in the "response to reviewers" section.

-A tracked changes document

- A clean version of the latest version of the manuscript

To upload your revision, please click the link below.

https://plantdirect.msubmit.net/cgi-bin/main.plex?el=A6Lr6lP4A2iBI7I7A9ftdtA2ml6Z5uEa4CQGa95oyFgZ

In order to provide as timely a service as possible, we ask that your revision is resubmitted within three months after receipt of this request. If an extension is needed, please send a request, along with a brief explanation, to the editorial office at plantdirect@wiley.com .

Thank you very much for giving us an opportunity to review your work. I look forward to receiving the next version.

Sincerely,

Jyothilakshmi Vadassery

- Editor comments:

Both the reviewers have appreciated the MS, but raised a few questions on the replicates used. Reviewer 2 has also pointed to different processing pipelines within the MS and multiple overlapping raw data submissions to the same repository. Kindly address all the questions of both reviewers, and we look forward to seeing the revised MS

-- Reviewer comments:

Reviewer #1:

The manuscript by Saur et al. details the development of an untargeted proteomics approach to identify ADP-ribosylated (ADPr) proteins in plants, with a focus on host targets of ADP-ribosyltransferase effectors from plant-pathogenic bacteria (AvrRpm1, HopF2, and HopU1). ADP-ribosylation is an important post-translational modification (PTM) that impacts protein activity and has roles in biotic and abiotic stress responses in plants and animals. Previous studies have focused on specific host targets of these ADP-ribosyltransferase effectors that, with the exception of GRP7, were first identified through other approaches. To my knowledge, this is one of the first, if not the first, reports of using a modern untargeted proteomics approach to identify ADPr-modified proteins in plants.

ADP-ribosylation is a technically challenging PTM to study, owing to the relatively low abundance of the modification, requiring enrichment prior to analysis, as well as its chemical lability both in the cell and during sample processing and subsequent LC-MS/MS analyses. Furthermore, the diversity of amino acid residues (Asp/Glu/Ser/Thr/Tyr/pSer/His/Lys/Asn/Cys) that can be modified by ADPr makes precise localization of the PTM during data analyses even more difficult.

Saur et al. tackle this challenge by optimizing methodology for sample processing, enrichment of ADPr-modified proteins, and LC-MS/MS acquisition parameters to increase the AGC and ion fill time when diagnostic ions from ADPr fragmentation are detected. The authors identified ADPr-modified peptides from known and assumed targets of AvrRpm1 (RIN4, NOI4, and NOI12), and the modification occurred at sequence-equivalent locations in these proteins' respective NOI domains. The authors identified additional proteins that were specifically enriched using the ADPr-binding domain Af1521, indicating they are also ADP-ribosylated. An ADPr-modified peptide from the MAMI protein was identified in Arabidopsis samples, and AvrRpm1-dependent ADP-ribosylation of MAMI was confirmed using transient expression in N. benthamiana. Another enriched protein, PLDGAMMA3 was confirmed to be ADPr-modified when co-expressed with AvrRpm1 in N. benthamiana. The identification of MAMI and PLDGAMMA3 as new targets of AvrRpm1 will facilitate future investigations into the role of these proteins during plant immune responses. The authors extended the methodology to HopU1 and could identify a previously known ADPr-modified peptide from GRP7, an established target of HopU1.

Overall, I believe this manuscript presents a viable approach to identify ADP-ribosylation targets of pathogen effectors in plants. This is supported by the identification of known effector-modified target sites on host proteins. The methodology is reasonable, the experimental controls are sufficient, and the conclusions are sound. One drawback is that the minimal biological replication prevents statistical analysis of protein enrichment for many of the ADPr pulldown experiments, but I understand that the focus was more on method optimization. It is somewhat disappointing that not many more protein targets and modified peptides were identified from what was already known, but this could be due to technical limitations and/or the nature of the effectors that were studied. I note that there were not many unique bands on most of the SDS-PAGE immunoblots probing for ADPr-proteins, suggesting that the effectors truly could have a limited number of host targets, or the targets are very low abundance and/or not efficiently extracted from plant tissue using this protocol. Regardless, I believe that this paper represents an important contribution to studying ADP-ribosylation in plants. I have a few comments below that I hope the authors find constructive. I support acceptance of this manuscript with relatively minor revisions.

Table 1:

1. In Table 1, the protein list and first 3 columns of PSM data come from Supp. Data 1. The PSM ratio data comes from Supp. Data 4. NOI4 and NOI12 are noticeably absent from Table 1. The table legend could be clearer on where the data came from and the replicates used. There are other proteins enriched in Supp. Data 4, the Af1521 Macro vs G42E experiment. For example, NOI4 (AT5G55850) and PapD-like superfamily protein (AT5G55850); should those be reported in Table 1 as well? Was there a rationale for choosing to report only proteins with >10 summed PSMs? Also, It might be worthwhile to report the unique peptides identified for each protein in parentheses after the PSM counts.

Methods:

I think that more information could be added to the methods, and the methods could be organized differently. Specifically:

2. For the "Enrichment of ADP-ribosylated proteins", what was the plant sample protein input for incubation with GST-Af1521Macro (and G42E)? If different inputs were used in different experiments, this should be noted.

3. More information is needed for the pre-clearing of samples with the HiTrap desalting. This might be better in its own section of the methods. How much protein sample was loaded onto the columns? What system was used for desalting (chromatography, peristaltic pump, or syringe)? Flow rate? Elution volume? Currently this part of the workflow is only mentioned on lines 184-185, in the "Enrichment of ADP-ribosylated proteins" section.

4. Move the LC-MS/MS methods from supplemental methods to the main methods. What was the peptide load injected into the MS? If different inputs were used in different experiments, this should be noted.

5. It makes sense to move the "In vitro ADP-ribosylation assay" section to supplemental methods, as this assay was used only for a supplemental figure experiment that was referenced in the discussion.

Discussion:

6. It is somewhat surprising that the authors did not identify more ADPr-modified proteins by LC-MS/MS, especially when considering the signal in Fig 1C (last lane). There is not much discussion by the authors on possible biological or technical reasons for this observation.

7. Based on the knowledge gained during this project, are there any potential improvements to this methodology that might result in greater coverage of ADPr modifications in plants? For example, would PARG treatment have facilitated the identification of more ADPr sites? As this is primarily a method development paper, addressing this in the discussion would be useful for readers.

8. Was there any benefit for performing pulldowns with higher concentrations of Af1521 Macro domain? It appears that the ~26 kDa band is still present in the supernatant fractions. Would more than 5 ug of the Macro domain be beneficial for more efficient recovery of ADPr proteins? Commenting on this might be useful to others attempting to replicate similar experiments.

Reviewer #2:

In their publication "Untargeted proteomics identifies plant substrates of the bacterial-derived ADP2 ribosyltransferase AvrRpm1" Kaura, et al. adapt an enrichment strategy established in mammalian cells for a specific PTM to plant lysates. The authors show that their protocol can enrich for ADP-ribosylated proteins / peptides. The overall numbers lack behind the rates reported in other organisms. The publication gives limited insights into the biological relevance of newly discovered substrates.

While the acquisition strategy - DDA - is in general considered "untargeted", the follow up analysis is focusing on just a few (known) targets, rather than exploring the dataset as a whole.

The lack of replicate measurements as well as a streamlined analysis of the data makes major revisions necessary.

37 reference?

128 create sub-headings for better readability / overview

175 Amido black stain?

222 Elaborate on the other proteins detected in Dataset S2. Why were the runs searched and uploaded separately? Why is there no control included? Why are there no replicates for the different conditions / loadings? How was the overlap between conditions?

229 See 222, in addition: How was the overlap between triggered / untriggered?

266 Dataset S1 and 4 should be re-analysed together for a reliable quantitative comparison, preferably via LFQ / precursor ion quant in MaxQuant.

652 DS3 contains 3 raw files in 3 submissions, while DS8 contains 5 rawfiles in total. Please elaborate / rephrase

1. Are the experiments and analysis performed to an acceptable technical standard?

No - Within the manuscript, data (of the same type / source) is analysed with two fundamentally different processing pipelines. In addition to that, the raw data is spread out over 12 different submissions to the same repository.

Commonly, all data analysed within one manuscript is submitted as one data set to a repository (with respective meta data) and the same software suit (even the same version for separate runs) is used for analysis. Replicate runs are not submitted as single entries to a repository, but as sets.

When different SW is used within a publication, this is usually done in a comparative / additional way, to complement the results or benchmark the performance of pipelines for particular use cases. Performance and overlap should be evaluated.

No general overview is given of the dataset. How many protein groups were detected in general, how many quantified? How was the overlap between replicates? How was the overall intensity correlation?

The analysis sets a strong focus on identification rather than quantitative comparison. This is a (out)dated way of assessing MS data.

In addition, when quantifying, the authors use PSM counts, but also used dynamic exclusion when acquiring the data. This is not a reasonable combination: dynamic exclusion will exclude a particular

feature from being sequenced (submitted to MS2) to often - allowing for more depth of the analysis, by allowing more features to be fragmented. When on the (dynamic) exclusion list, an analyte will not produce PSMs during that time. This will in turn lead to a skewed ratio between number of PSMs and abundance.

For the analysis of coIP experiments by LC-MS this is commonly analysed in an enrichment analysis. Which proteins are enriched by the immobilized Af1521 Macro domain relative to the G42E control?

Raw data in DS 2 and 3 was searched separately, rather than as one dataset.

2. Are the experiments and analysis described in adequate detail in standard English?

Yes

3. Do the results support the conclusion?

Yes, in principle. The lack of replicates however puts reliability in question.

4. Does the manuscript and contents meet all ethical and research integrity standards?

No - As outlined in the „Experimental design and statistical rationale" section (L 103-126) experiments and / or LC-MS measurements were only performed in two replicates in some cases.

Figures 1 - 3 (and Suppl Fig S1, 2, 4): Assigned ion labels and m/z of ions are only readable in the source PDF - when zoomed in. This is an issue when exporting from the MQ viewer. Please provide additional zoom-ins of the relevant sections of the spectra. Consider alternative viewers. As suggested e.g. by MCP (https://www.mcponline.org/annotated-spectra), upload to MS-Viewer, provide the (full) combined folder or use https://github.com/wenbostar/PDV.

Formatting:

Use subscript when appropriate, esp. for log2(subscript) / log10(subscript), or C18(subscript)

Use non-breaking spaces between value and unit to improve readability.

Explain abbreviations at first use - e.g. Dex (L 108)

Decision Letter Round 2:

August 11, 2025

Plant Direct
Open Access

Dr. Lennart Wirthmueller

Leibniz-Institut fur Pflanzenbiochemie

Biochemistry of Plant Interactions

Weinberg 3

06120

Halle 14195

Germany

RE: Untargeted proteomics identifies plant substrates of the bacterial-derived ADP-ribosyltransferase AvrRpm1

Dear Dr. Wirthmueller:

Thank you for submitting to Plant Direct. All required reviews have been returned and we have now finished our evaluation of your manuscript. In light of the reviewers' and editor's comments, further revisions are needed before the paper can be accepted for publication in Plant Direct.

Please view the editors' and reviewers' comments below and use their suggestions as a guide while you work on your revision.

When uploading the revised version of this article, please be sure to include the following:

-A word document that contains your response to the reviewers. You should respond to each reviewer comment and note the changes made to the manuscript. If you do not agree with a reviewer's comment and choose not to make a suggested revision, please explain why. Please try to provide as complete an answer as possible to each reviewer's criticisms.

-A tracked changes document with each change highlighted

- A clean version of the latest version of the manuscript

Kindly upload the raw proteomic data on servers recommended by reviewer as it will enhance its value and be useful to many other readers

To upload your revision, please click the link below.

https://plantdirect.msubmit.net/cgi-bin/main.plex?el=A6Lr1lP4B7iBI6I6A9ftdtA2ml6Z5uEa4CQGa95oyFgZ

In order to provide as timely a service as possible, we ask that your revision is resubmitted within three months after receipt of this request. If an extension is needed, please send a request, along with a brief explanation, to the editorial office at plantdirect@wiley.com .

Please note that, in addition to publishing reviewer comments, the author's responses to review comments will also be published alongside the final version of the paper. If you would not like the author's responses to be published, please contact the editorial office at plantdirect@wiley.com .

Thank you very much for giving us an opportunity to review your work. I look forward to receiving the next version.

Sincerely,

Jyothilakshmi Vadassery

Gustavo MacIntosh

Editor, Plant Direct

--- Editor comments:

Kindly upload the raw proteomic data on servers recommended by reviewer as it will enhance its value and be useful to many other readers

--- Reviewer comments:

Reviewer #2:

The authors have taken the reviewers' comments seriously and have made a good effort to address them in a reasonable time with acceptable depth. The response is comprehensive. The authors have made substantial changes to improve the manuscript, esp. by streamlining their analysis pipeline.

The addition of more replicates is a significant improvement. The switch to one tool for all analyses and the re-analysis of existing datasets are positive steps. The authors' responses are detailed and provide a clear, (mostly) comprehensible rationale for most changes. Some concerns still remain, particularly regarding the sharing of raw and processed data (simply due to technical issues).

Data can be provided to https://www.proteomexchange.org/ via various other ways, e.g. via https://repository.jpostdb.org/ (fully web based) - "jPOST is a certificated member of ProteomeXchange Consortium and jPOSTrepo provides official ProteomeXchange Identifiers to projects stored in our repository."

https://msviewer.ucsf.edu/prospector/cgi-bin/msform.cgi?form=msviewer provides full spectral access, is compatible with MaxQuant outputs and also web based.

Besides that, there are platforms, such as http://zenodo.org/ or http://figshare.com/ allowing to share big data in a straight forward way.

Please make use of those (established, widely accepted) alternatives.

I recommend accepting the manuscript with minor revisions, focusing on / addressing the sharing of their data adequately.

Decision Letter Round 3:

October 3, 2025

Dr. Lennart Wirthmueller

Leibniz-Institut fur Pflanzenbiochemie

Biochemistry of Plant Interactions

Weinberg 3

06120

Halle 14195

Germany

MSID: 2024-01649R2

MS TITLE: Untargeted proteomics identifies plant substrates of the bacterial-derived ADP-ribosyltransferase AvrRpm1

Dear Dr. Lennart Wirthmueller:

I am pleased to inform you that your manuscript "Untargeted proteomics identifies plant substrates of the bacterial-derived ADP-ribosyltransferase AvrRpm1" has been accepted for publication in Plant Direct.

Your article will appear online in the next available issue of Plant Direct. To ensure your article gets published as quickly as possible, please pay attention to the steps detailed below. We have found that most of the delays happen at this stage, especially at the payment stage, so please respond as quickly as possible when prompted.

License Agreement: Once your article has been accepted it will move to Production and undergo admin and file checking - you may receive an email with any queries we have at this stage. When all required items are received by the publisher and queries resolved, the corresponding author will receive an email from Wiley's Author Services system which will ask them to log on at

https://authorservices.wiley.com/bauthor and will present them with the appropriate license for completion. Your article cannot be published until both the signed license agreement and payment of the article fee have been received.

Payment of the Open Access Article Publication Fee: All articles published in Plant Direct are fully open access: immediately and freely available to read, download and share. Plant Direct charges a publication fee to cover the publication costs. The corresponding author for this manuscript should have already received a quote from the payments team (cs-openaccess@wiley.com) with the estimated article publication fee; please email cs-openaccess@wiley.com if this has not been received. The corresponding author should log on to the Wiley Author Services site, where the publication fee can be paid by credit card or an invoice. Pro Forma can also be requested. Payment of the publication charge must be received before the article will be published online.

Proofs: You will have the opportunity to look over your paper once more when you receive the author proofs for your article. The proofs will be with you in approximately two weeks. Please note that, in addition to publishing reviewer comments, the author's responses to review comments will also be published alongside the final version of the paper. If you would not like the author's responses to be published, please contact the editorial office at plantdirect@wiley.com .

Promotion of your article: You can help your research get the attention it deserves! Check out Wiley's free Promotion Guide for best-practice recommendations for promoting your work at www.wileyauthors.com/eeo/guide. And learn more about Wiley Editing Services which offers professional video, design, and writing services to create shareable video abstracts, infographics, conference posters, lay summaries, and research news stories for your research at www.wileyauthors.com/eeo/promotion.

Thank you again for your contribution to Plant Direct. If you have any questions, feel free to contact the editorial office at plantdirect@wiley.com .

Sincerely,

Jyothilakshmi Vadassery

Gustavo MacIntosh

Editor, Plant Direct

Response Letter Round 1:

Response to reviewers‘ comments - Editor comments: Both the reviewers have appreciated the MS, but raised a few questions on the replicates used. Reviewer 2 has also pointed to different processing pipelines within the MS and multiple overlapping raw data submissions to the same repository. Kindly address all the questions of both reviewers, and we look forward to seeing the revised MS We thank the two reviewers and the editor for their critical feedback and their constructive comments on our manuscript. We provide our answers in the point-by-point response below. --- Reviewer comments: Reviewer #1: The manuscript by Saur et

al. details the development of an untargeted proteomics approach to identify ADP-ribosylated (ADPr) proteins in plants, with a focus on host targets of ADP-ribosyltransferase effectors from plant-pathogenic bacteria (AvrRpm1, HopF2, and HopU1). ADP-ribosylation is an important posttranslational modification (PTM) that impacts protein activity and has roles in biotic and abiotic stress responses in plants and animals. Previous studies have focused on specific host targets of these ADPribosyltransferase effectors that, with the exception of GRP7, were first identified through other approaches. To my knowledge, this is one of the first, if not the first, reports of using a modern untargeted proteomics approach to identify ADPr-modified proteins in plants. ADP-ribosylation is a technically challenging PTM to study, owing to the relatively low abundance of the modification, requiring enrichment prior to analysis, as well as its chemical lability both in the cell and during sample processing and subsequent LC-MS/MS analyses. Furthermore, the diversity of amino acid residues (Asp/Glu/Ser/Thr/Tyr/pSer/His/Lys/Asn/Cys) that can be modified by ADPr makes precise localization of the PTM during data analyses even more difficult. Saur et al. tackle this challenge by optimizing methodology for sample processing, enrichment of ADPrmodified proteins, and LC-MS/MS acquisition parameters to increase the AGC and ion fill time when diagnostic ions from ADPr fragmentation are detected. The authors identified ADPr-modified peptides from known and assumed targets of AvrRpm1 (RIN4, NOI4, and NOI12), and the modification occurred at sequence-equivalent locations in these proteins' respective NOI domains. The authors identified additional proteins that were specifically enriched using the ADPr-binding domain Af1521, indicating they are also ADP-ribosylated. An ADPr-modified peptide from the MAMI protein was identified in Arabidopsis samples, and AvrRpm1-dependent ADP-ribosylation of MAMI was confirmed using transient expression in N. benthamiana. Another enriched protein, PLDGAMMA3 was confirmed to be ADPr-modified when co-expressed with AvrRpm1 in N. benthamiana. The identification of MAMI and PLDGAMMA3 as new targets of AvrRpm1 will facilitate future investigations into the role of these proteins during plant immune responses. The authors extended the methodology to HopU1 and could identify a previously known ADPr-modified peptide from GRP7, an established target of HopU1. Overall, I believe this manuscript presents a viable approach to identify ADP-ribosylation targets of pathogen effectors in plants. This is supported by the identification of known effector-modified target sites on host proteins. The methodology is reasonable, the experimental controls are sufficient, and the conclusions are sound. One drawback is that the minimal biological replication prevents statistical analysis of protein enrichment for many of the ADPr pulldown experiments, but I understand that the focus was more on method optimization. It is somewhat disappointing that not many more protein targets and modified peptides were identified from what was already known, but this could be due to technical limitations and/or the nature of the effectors that were studied. I note that there were not many unique bands on most of the SDS-PAGE immunoblots probing for ADPr-proteins, suggesting that the effectors truly could have a limited number of host targets, or the targets are very low abundance and/or not efficiently extracted from plant tissue using this protocol. Regardless, I believe that this paper represents an important contribution to studying ADP-ribosylation in plants. I have a few comments below that I hope the authors find constructive. I support acceptance of this manuscript with relatively minor revisions. Thank you for the constructive feedback and appreciation of the technical challenges. Table 1: 1. In Table 1, the protein list and first 3 columns of PSM data come from Supp. Data 1. The PSM ratio data comes from Supp. Data 4. NOI4 and NOI12 are noticeably absent from Table 1. The table legend could be clearer on where the data came from and the replicates used. There are other proteins enriched in Supp. Data 4, the Af1521

Macro vs G42E experiment. For example, NOI4 (AT5G55850) and PapD-like superfamily protein (AT5G55850); should those be reported in Table 1 as well? Was there a rationale for choosing to report only proteins with >10 summed PSMs? Also, It might be worthwhile to report the unique peptides identified for each protein in parentheses after the PSM counts. To provide a more robust data basis for Supplementary Dataset (DS) 1, we have performed two additional experiments comparing Af1521-enriched candidate proteins from Col-0, HopF2, and AvrRpm1 lines. We have re-analyzed the data in MaxQuant as the use of two different types of processing software was another valid point of criticism by Reviewer 2. We have included label-free quantification (LFQ) in MaxQuant, but realized that only three of the proteins listed in the first version of Table 1 can be quantified by LFQ with default settings. This is due to the low abundance of some of the proteins, the stringent criteria of LFQ in MaxQuant, and heterogeneity of the individual experiments (change of column manufacturer on the MS in 2024, experiments performed over a timeframe of three years). We included the LFQ analysis in the revised Table 1, which is now based on four independent experiments, but kept PSM (now number of MS2 scans identified by MaxQuant) as a basis for including proteins in the table. As Mascot/Proteome Discoverer and MaxQuant have different ways of assigning peptides to protein groups the numbers have slightly changed. We kept the stringent criterion that to be included in Table 1, a protein must be identified by at least 10 collective MS2 scans in AvrRpm1, but must not be detected in any of the Col-0 and HopF2 samples. For DS4 (enrichment with Af1521 Macro vs. G42 control) we performed one additional experiment so that the analysis is now based on three individual experiments. Although the proteins highlighted in Table 1 are not entirely identical to the top hits of DS4, they overall show a robust overlap. We now provide a more detailed analysis of DS4 in lines 361-371 of the revised manuscript. To keep Table 1 comprehensive, we decided against including the identified peptides in the table. Instead, we provide this information by including the evidence.txt file from the MaxQuant analysis in the Excel files. We also report the number of experiments in the table header. Methods: I think that more information could be added to the methods, and the methods could be organized differently. Specifically: 2. For the "Enrichment of ADP-ribosylated proteins", what was the plant sample protein input for incubation with GST-Af1521Macro (and G42E)? If different inputs were used in different experiments, this should be noted. The starting material was 1.5-2 g for samples from transient expression in N. benthamiana and 2-4 g for Arabidopsis samples. Samples that we compared directly had the same amount of starting leaf material. We now mention this in line 163 of the methods section. 3. More information is needed for the pre-clearing of samples with the HiTrap desalting. This might be better in its own section of the methods. How much protein sample was loaded onto the columns? What system was used for desalting (chromatography, peristaltic pump, or syringe)? Flow rate? Elution volume? Currently this part of the workflow is only mentioned on lines 184-185, in the "Enrichment of ADP-ribosylated proteins" section. Thank you for the suggestion. We now describe the pre-clearing in a separate paragraph of the methods section (lines 190-196). 4. Move the LC-MS/MS methods from supplemental methods to the main methods. What was the peptide load injected into the MS? If different inputs were used in different experiments, this should be noted. We have moved the LC-MS/MS methods to the main methods as suggested. The peptide load was not quantified prior to the LC-MS/MS but was assessed by initially running a 1 μL aliquot of each sample from which we extrapolated the maximal sample volume that can be applied to the LC column. We have included this information in lines 219-220 of the methods section. 5. It makes sense to move the "In vitro ADP-ribosylation assay" section to supplemental methods, as this assay was used only

for a supplemental figure experiment that was referenced in the discussion. We agree and we have moved this section as suggested. Discussion: 6. It is somewhat surprising that the authors did not identify more ADPr-modified proteins by LC-MS/MS, especially when considering the signal in Fig 1C (last lane). There is not much discussion by the authors on possible biological or technical reasons for this observation. We agree that there is an apparent discrepancy between results from the western blot and the detection of ADPr-modified peptides by LC-MS/MS. Possible reasons are: i) While the antibody detects mono-ADPr as well as oligo- and poly-ADPr chains, proteomics will only detect mono-ADPr peptides (unless the samples have been efficiently processed with a PARG enzyme, see also response to Reviewer 2 below). It appears possible that mono-ADP- ribosylation catalyzed by AvrRpm1 can serve as a starting point for poly-ADP-ribosylation by plant enzymes, but we have not investigated this further. ii) The chemical instability of ADP-ribosylation on Glu and Asp, particularly under basic pH conditions, might lead to partial loss of the modification during tryptic digest. Alternative proteases and PAGE/blotting systems that are compatible with more neutral or slightly acidic pH values have recently been suggested as a point for improvement (Longarini & Matic, 2024, included as additional citation). As these possibilities remain very speculative, we have not included them in the revised manuscript. However, we can discuss these points in case the reviewers consider them helpful rather than too speculative. 7. Based on the knowledge gained during this project, are there any potential improvements to this methodology that might result in greater coverage of ADPr modifications in plants? For example, would PARG treatment have facilitated the identification of more ADPr sites? As this is primarily a method development paper, addressing this in the discussion would be useful for readers. We agree with Reviewer 1 that this is a valid point for the discussion that was also raised by Reviewer 2. We have included it in lines 466-480 of the revised discussion. In principle, a treatment with PARG should facilitate detection of ADP-ribosylated peptides from poly-ADP-ribosylated plant proteins but not from effector-catalyzed mono-ADP-ribosylation. As there is currently no evidence suggesting that ADPribosylating effectors are ‘poly-enzymes’, we did not include a PARG treatment in the method. Another reason is that, if not carefully titrated, the addition of PARG can even lead to hydrolysis of ADPr from peptides, now mentioned in lines 474-477 with the respective citations. With respect to detection of ADPr sites from poly-ADP-ribosylated peptides, in our experience a simple transfer of methods described for mammalian cell cultures to samples from plant extracts is less straightforward than initially assumed. We address this point in lines 466-480 of the revised discussion. 8. Was there any benefit for performing pulldowns with higher concentrations of Af1521 Macro domain? It appears that the ~26 kDa band is still present in the supernatant fractions. Would more than 5 ug of the Macro domain be beneficial for more efficient recovery of ADPr proteins? Commenting on this might be useful to others attempting to replicate similar experiments. We did not thoroughly test a broad range of Af1521 amounts for the affinity enrichment and we did not include a ‘supernatant’ fraction after the pulldown in Fig. 1C. In panel C we compare the ADPr western blot signals between total extracts of Col-0 and Dex-treated AvrRpm1 lines on the left side. The right side of the blot shows the difference of including the Sephadex G-25 desalting step in the protocol. The choice for 2.5 μg was based on the analysis of Supplementary Datasets S2 and S3. When DS2 and DS3 are analyzed together with respect to different amounts of Af1521, the two 1 μg samples identified 7 ADPr sites (avg. score of 75). There was an apparent improvement when using 2.5 μg Af1521 (12 ADPr sites, avg. score of 113) that did not further improve when using 5 μg (11 ADPr sites, avg. score of 110). We now explain the admittedly limited basis for performing subsequent experiments with 2.5 μg Af1521 in lines 326-328 and provide the

comparison as Supplementary Table S2. Reviewer #2: In their publication "Untargeted proteomics identifies plant substrates of the bacterial-derived ADP2 ribosyltransferase AvrRpm1" Kaura, et al. adapt an enrichment strategy established in mammalian cells for a specific PTM to plant lysates. The authors show that their protocol can enrich for ADP-ribosylated proteins / peptides. The overall numbers lack behind the rates reported in other organisms. The publication gives limited insights into the biological relevance of newly discovered substrates. While the acquisition strategy - DDA - is in general considered "untargeted", the follow up analysis is focusing on just a few (known) targets, rather than exploring the dataset as a whole. The lack of replicate measurements as well as a streamlined analysis of the data makes major revisions necessary. To address the point of lacking replicate measurements, we have performed additional experiments as follows: DS1 -> two additional experiments, now based on four experiments DS4 -> one additional experiment, now based on three experiments DS6 -> one additional experiment, now based on three experiments 37 reference? In our opinion the cited publication supports the statement made in this sentence. 128 create sub-headings for better readability / overview Thank you for the suggestion. This has been changed. 175 Amido black stain? We use this dye as a non-specific protein stain for Western blot membranes, as described for example here: https://en.wikipedia.org/wiki/Amido_black_10B 222 Elaborate on the other proteins detected in Dataset S2. Why were the runs searched and uploaded separately? Why is there no control included? Why are there no replicates for the different conditions / loadings? How was the overlap between conditions? Please see next point. 229 See 222, in addition: How was the overlap between triggered / untriggered? The aim of these experiments (DS S2 and S3) was to optimize conditions for detecting the ADPribosylation sites on RIN4 with the hope that these conditions would also facilitate identification of other (incl. endogenous) modification sites. In order to exclude variations from multiple sample preparations, all samples were derived from one AvrRpm1-expressing plant extract that was limited in volume by the maximal capacity of the HiTrap column used for sample pre-clearing. Although this approach excludes variation from plant sample processing, we acknowledge that technical differences between the six subsequent LC-MS/MS runs could affect our interpretation of the results. We therefore re-analyzed DS2 and DS3 together in MaxQuant. To compare all experiments side-by-side based on LFQ, we included all protein groups that had three LFQ intensity values assigned in at least one of the two DS. The Pearson correlations of LFQ intensities between single runs ranged from 0.85 – 0.98 (new Supplementary Figure S2 B). Therefore, we consider them overall comparable. In DS2 there were 133 protein groups. In DS3, seven additional proteins were quantified. Three of them (AT1G47570, AT5G02870, AT2G45640) were detected in all three experiments of DS3 but not in DS2 and are significant based on a two-sided t-test. We show this in a volcano plot in the new Supplementary Fig. S2 A. For the seven protein groups quantified in DS3 but not in DS2 we did not detect any ADP-ribosylation sites although the RIN4 homologs NOI10 and/or NOI11 are in the list. protein groups quantified by LFQ in DS3 but not DS2 protein identifiers protein name AT1G47570.1 RING/U-box superfamily protein AT5G02870.1 RIBOSOMAL LARGE SUBUNIT 4 AT5G48657.1; AT3G07195.1 NOI10; NOI11 AT2G45640.1 ATSAP18 AT4G17560.1 RIBOSOMAL PROTEIN BL19CZ AT2G29440.1 GSTU6 AT5G07090.1;AT2G17360.1;AT5G58420.1 RIBOSOMAL PROTEIN S4 class The major difference between the two methods used for DS2 and DS3 is qualitative. Although the standard method (DS2) identified more potential ADP-ribosylation sites, all other qualitative measures improved with the triggering method (DS3). Particularly, the average localization probability of the modification (0.63 vs. 0.43) and the percentage of high-confidence localizations (38.5 vs. 5.9) substantially increased. In

conclusion, the standard method reports an apparently higher number of ADPribosylation sites, but these additional sites are low-confidence identifications. The triggering method identifies ADP-ribosylation sites with higher confidence and more precise localization. The radar plots shown below are also included in Supplementary Fig. S2 C and data for these comparisons is provided in the new Supplementary Table S1. # proteins = number of ADP-ribosylated proteins ADPr sites = number of ADP-ribosylation sites score = average Andromeda score of the ADP-ribosylated peptides loc. score = average localization score for the modification loc. probability = average localization probability for the modification % ADPr marker = percentage of peptides with ADPr marker ions % loc. prob. > 0.99 = percentage of peptides with a high-confidence localization score >0.99 for the modification 266 Dataset S1 and 4 should be re-analysed together for a reliable quantitative comparison, preferably via LFQ / precursor ion quant in MaxQuant. As mentioned in the response to Reviewer 1 we have performed this analysis based on LFQ. We found that precursor data for several proteins like MAMI were not sufficient to be included in the quantification. We think that this is in part explained by the heterogeneity of the data from individual experiments over several years and technical changes on the instrument. As proteins like MAMI are clearly a substrate of AvrRpm1 (Fig. 3), an analysis entirely based on LFQ might provide a statistically more robust analysis but would miss relevant identifications. For LFQ-based quantification we would ideally have kept variations in plant growth chambers as small as possible and would have preferred to analyze all samples as one set by LC-MS/MS. However, as our analysis based on PSM (now MS2 scan counts) was successful in identifying known and novel substrates of AvrRpm1, we decided against a complete repetition of experiments for DS1 and DS4 as this would also have been difficult to achieve within the timeframe for the revision. We have included the results of the LFQ analysis in the new Supplementary Figures S1 and S4 and describe them in lines 281-288 (DS 1) and 365-371 (DS 4). 652 DS3 contains 3 raw files in 3 submissions, while DS8 contains 5 rawfiles in total. Please elaborate / rephrase Thank you for raising this discrepancy. In order to maximize chances of identification, we had included the two raw files from DS4 where we also enriched ADP-ribosylated peptides from Dex-induced AvrRpm1 lines in addition to the three raw files from DS3. As the re-analysis for peptides with ADP-ribosylation and phosphorylation was inconclusive and negative data from proteomics are no proof of absence, we decided against including this dataset again in the revised version of the manuscript. 1. Are the experiments and analysis performed to an acceptable technical standard? No - Within the manuscript, data (of the same type / source) is analysed with two fundamentally different processing pipelines. In addition to that, the raw data is spread out over 12 different submissions to the same repository. Commonly, all data analysed within one manuscript is submitted as one data set to a repository (with respective meta data) and the same software suit (even the same version for separate runs) is used for analysis. Replicate runs are not submitted as single entries to a repository, but as sets. We apologize if the submission as one set is a commonly known procedure that we did not follow. The reason for individual submissions was the intuitive preference to clearly associate the detected ADPribosylation sites with the corresponding raw files. As stated above, we have re-analyzed DS2 and DS3 together, including LFQ, and now provide a more extensive comparison in the new Supplementary Fig. S2 and Supplementary Table S1. In Table S1, the ADP-ribosylated peptides are separated between DS2 and DS3 based on the 'Best score raw file' column. We agree with Reviewer 2 that the use of two separate processing pipelines was not appropriate. This was due to two authors performing the analysis. The revised manuscript is now entirely based on analysis with MaxQuant/Perseus. For this reason, we also had to remove the previous Supplementary Figures S1

and S2 that were based on exporting peak lists from Proteome Discoverer. We noted that the small peaks indicating ADP-ribosylation on the residue N-terminal to the Trp are not assigned with default settings in MaxQuant. As the intensity of these peaks was small with respect to the signal-to-noise ratio, we prefer to keep the original representation of ADP-ribosylation sequence windows above the ion series. The removal of Supplementary Figures S1 and S2 does not change the interpretation of our results as the reported ADP-ribosylation sites are still supported by the data from the revised manuscript. The combined analysis of DS2 and DS3 is now provided on the shared drive (see end of our response). When different SW is used within a publication, this is usually done in a comparative / additional way, to complement the results or benchmark the performance of pipelines for particular use cases. Performance and overlap should be evaluated. As stated above we have performed this analysis and describe the results in the revised manuscript. No general overview is given of the dataset. How many protein groups were detected in general, how many quantified? How was the overlap between replicates? How was the overall intensity correlation? The analysis sets a strong focus on identification rather than quantitative comparison. This is a (out)dated way of assessing MS data. We now report the number of protein groups, number of quantified proteins by MS2 scans vs. LFQ, and percent overlap in the revised manuscript. for DS1 in lines 276-288 for DS2+DS3 in lines 306-315 for DS4 in lines 361-371 For DS2 and DS3 that we compared directly with respect to performance of the two methods, we also calculated the pairwise Pearson correlation score in Supplementary Fig. S2 B. In addition, when quantifying, the authors use PSM counts, but also used dynamic exclusion when acquiring the data. This is not a reasonable combination: dynamic exclusion will exclude a particular feature from being sequenced (submitted to MS2) to often - allowing for more depth of the analysis, by allowing more features to be fragmented. When on the (dynamic) exclusion list, an analyte will not produce PSMs during that time. This will in turn lead to a skewed ratio between number of PSMs and abundance. This is a valid point. As described above, basing the analysis entirely on the more stringent MaxQuant LFQ would miss protein identifications (e.g. MAMI) that we consider worth reporting and thus have investigated by complementary methods. Therefore, for DS1 and DS4, we kept the quantification based on number of MS2 scans as a primary basis for analysis but we do report results from LFQ alongside in Table 1, and Supplementary Figs. S1, S2, S4. While dynamic exclusion has been reported to skew the ratio of PSMs/abundance, we consider our stringent filtering criteria (≥ 10 vs. 0 MS2 scans) and the increased number of replicate samples sufficient to prevent reporting inappropriate numbers of false positives. For the analysis of coIP experiments by LC-MS this is commonly analysed in an enrichment analysis. Which proteins are enriched by the immobilized Af1521 Macro domain relative to the G42E control? Raw data in DS 2 and 3 was searched separately, rather than as one dataset. In DS2 and DS3 the enrichment was only performed with the Af1521 Macro domain and we provide a volcano plot of significantly enriched proteins in Supplementary Fig. S2 and describe differences between the datasets in lines 306-315. For DS1 and DS4 we have now included an enrichment analysis based on LFQ in Supplementary Figs. S1 and S4 and also mention differences between the datasets as stated above. 2. Are the experiments and analysis described in adequate detail in standard English? Yes 3. Do the results support the conclusion? Yes, in principle. The lack of replicates however puts reliability in question. We have performed the additional replicates for DS1 (comparison Col-0/HopF2/AvrRpm1), DS4 (comparison Af1521 vs. G42E), and DS6 (identification of the ADP-ribosylated sequence window from PLDG3). The results from DS5 (ADP-ribosylated sequence of MAMI) were confirmed by complementary methods in Fig. 3 (western blots) with three independent experiments. In addition,

we now mention in lines 380- 384 that the enrichment of ADP-ribosylated proteins from N. benthamiana leaves over-expressing PLDGAMMA3-HA and AvrRpm1-Flag also identified ADP-ribosylated peptides from N. benthamiana RIN4 and MAMI homologs that further corroborate the ADP-ribosylated peptides identified in experiments from Arabidopsis. The results from DS6 are still based on two experiments that both identified GRP7. The only intention here was to test the method on another ADP-ribosylating T3E. 4. Does the manuscript and contents meet all ethical and research integrity standards? No - As outlined in the „Experimental design and statistical rationale" section (L 103-126) experiments and / or LC-MS measurements were only performed in two replicates in some cases. We hope that we addressed this valid point of criticism above. Figures 1 - 3 (and Suppl Fig S1, 2, 4): Assigned ion labels and m/z of ions are only readable in the source PDF - when zoomed in. This is an issue when exporting from the MQ viewer. Please provide additional zoom-ins of the relevant sections of the spectra. Consider alternative viewers. As suggested e.g. by MCP (https://www.mcponline.org/annotated-spectra), upload to MS-Viewer, provide the (full) combined folder or use https://github.com/wenbostar/PDV. We have uploaded the raw data including the complete MaxQuant output to our shared MS OneDrive for review. We have chosen this option as i) our IT team was not able to provide us with the required Java runtime environment needed for the latest version of the PRIDE submission tool in time, and ii) the complete raw dataset is now ~74 GB and we consider it more economical if it can be reviewed first and then uploaded as a final version to PRIDE. As our institute only allows sharing folders on MS OneDrive with specific email addresses, I had to create the following account for anonymous review. Reviewers can log into the account under https://account.proton.me/mail email address: BPI_share@proton.me password: jDOn)jd92#ehH3JJ6 In the inbox the reviewers can find the invitation link to the shared MS OneDrive with all Supplementary Datasets as .zip files. Access to the shared folder may have to be confirmed by a code that will be sent to the same email address. Formatting: Use subscript when appropriate, esp. for log2(subscript) / log10(subscript), or C18(subscript) Use non-breaking spaces between value and unit to improve readability. Explain abbreviations at first use - e.g. Dex (L 108) We have addressed these points in the version of the revised manuscript without track changes.

Response Letter Round 2:

Response to reviewers‘ comments

- Editor comments:

Kindly upload the raw proteomic data on servers recommended by reviewer as it will enhance its value

and be useful to many other readers

-- Reviewer comments:

Reviewer #2:

The authors have taken the reviewers' comments seriously and have made a good effort to address them

in a reasonable time with acceptable depth. The response is comprehensive. The authors have made

substantial changes to improve the manuscript, esp. by streamlining their analysis pipeline.

The addition of more replicates is a significant improvement. The switch to one tool for all analyses and

the re-analysis of existing datasets are positive steps. The authors' responses are detailed and provide a

clear, (mostly) comprehensible rationale for most changes. Some concerns still remain, particularly

regarding the sharing of raw and processed data (simply due to technical issues).

Data can be provided to https://www.proteomexchange.org/ via various other ways, e.g. via

https://repository.jpostdb.org/ (fully web based) - "jPOST is a certificated member of ProteomeXchange

Consortium and jPOSTrepo provides official ProteomeXchange Identifiers to projects stored in our

repository."

https://msviewer.ucsf.edu/prospector/cgi-bin/msform.cgi?form=msviewer provides full spectral access, is

compatible with MaxQuant outputs and also web based.

Besides that, there are platforms, such as http://zenodo.org/ or http://figshare.com/ allowing to share

big data in a straight forward way.

Please make use of those (established, widely accepted) alternatives.

I recommend accepting the manuscript with minor revisions, focusing on / addressing the sharing of their

data adequately.

Response

Thank you for the assessment. We have uploaded the complete proteomics data including the

combined.zip folder from MaxQuant to the Proteomics Identifications Database. The data can be viewed

with the login/reviewer tokens shown in the table below. We will release the data and link them to the

manuscript’s DOI upon acceptance.

https://www.ebi.ac.uk/pride/login

Dataset Project accession Token

S1 PXD068442 SrUhKUIeWI4O

S2/S3 PXD068665 wFLhUCMWPIXc

S4 PXD068551 xL4O5nJY7LWc

S5 PXD068485 IkvMaS5vhvwq

S6 PXD068675 3EaNrD0u2OcZ

S7 PXD068739 8KM2q0harefe

Additional minor changes that we made to the manuscript are listed below.

- added ‘RIN4/NOI’ in line 321 to clarify that we refer to the peptides shown in Fig. 2
- corrected several wrong N. benthamiana protein identifiers in lines 380-382
- line 423, added the sentence ‘One possible explanation would be potential functional redundancy among the RIN4/NOI protein family members.’ to clarify that genetic redundancy within this protein family could also mask a disease phenotype in the rin4 mutant
